# Influence of pyridyl nitrogen’s position and hydrogen bonding interactions on antibacterial activities investigated by in vitro and in silico

**DOI:** 10.1038/s41598-025-09049-0

**Published:** 2025-07-30

**Authors:** Suma Ramachandra Gopady, Thripthi Nagesh Shenoy, Abdul Ajees Abdul Salam, Srikrishna Herga Damodar Kedlaya, Shashanka Puranika Kota, Anusha Prabhu, Yegneswaran Prakash Peralam, Srinivasulu Maddasani

**Affiliations:** 1 Department of Chemistry, Manipal Institute of Technology, Manipal Academy of Higher Education, Manipal, Karnataka 576 104 India; 2 Department of Applied Biosciences, Bhandarkars’ Arts and Science College, Kundapura, Karnataka India; 3 Manipal Institute of Applied Physics, Manipal Academy of Higher Education, Manipal, Karnataka 576104 India; 4 Centre for Microfluidics, Biomarkers, Photoceutics and Sensors (µBioPS), Department of Biotechnology, Manipal Institute of Technology, Manipal Academy of Higher Education, Manipal, Karnataka 576 104 India; 5 Department of Microbiology, Kasturba Medical College, Manipal Academy of Higher Education, Manipal, Karnataka 576104 India

**Keywords:** Schiff’s base, Pyridine derivatives, Hydrogen bonding interactions, Antibacterial activity, Paper disc diffusion method, in vitro, in silico, Microbiology, Chemistry

## Abstract

Pyridine derivatives are multifunctional materials with various biological activities viz., antibacterial, antiviral, anticancer, antihistamine, etc. and are also the building blocks in supramolecular chemistry. Schiff’s base compounds are known for their diversified applications / functionalities. A Schiff base with a pyridine moiety in its molecular structure is anticipated with potential biological activity. In order to study the effect of position of a pyridyl nitrogen on the antibacterial activity, two Schiff’s bases containing pyridine moiety viz., 3-chloro-4-fluoro-N-((pyridin-3-yl)methylene)benzenamine (3Py) and 3-chloro-4-fluoro-N-((pyridin-4-yl)methylene)benzenamine (4Py), were prepared by condensing 4-fluoro-3-chloro-aniline with 3-pyridinecarboxaldehyde and 4-pyridinecarboxaldehyde. Pyridine derivatives form a hetero synthon type intermolecular hydrogen bonding interactions with substituted benzoic acids. The lower homologues of 4-*n*-alkyloxybenzoic acids (*n*OBAs, ethyl, propyl and butyl homologues) were treated with the pyridine derivatives, 3Py and 4Py and were observed to be involved in intermolecular hydrogen bonding (HB) interactions. The pyridine derivatives (3Py and 4Py), benzoic acid derivatives and their 1:1 molar ratio complex were subjected to antibacterial activities through in vitro and in silico techniques. The antibacterial activities of the pristine compounds and their HB complexes against Gram-positive, *Staphylococcus aureus* and Gram-negative, *Escherichia coli* bacteria were studied by paper disc diffusion method. The antibacterial activity of the compounds is evaluated for their interactions with the key enzymes viz., *E. coli Mur*B and *S. aureus* GyraseB by in silico technique. The pyridyl nitrogen at 4th position of the Schiff base (4Py) showed effective inhibition towards the *S. aureus* whereas the pyridyl nitrogen at 3rd position (3Py) of the Schiff base showed effective inhibition towards the *E. coli*. The increase in alkyl chain length of carboxylic acids was found to decrease the antimicrobial activity in the pristine homologues. The HB interactions between 3Py and *n*OBA showed different antibacterial activities compared to that between 4Py and *n*OBA against *E. coli* and *S. aureus*. The experimental results were supported by molecular docking, molecular dynamics simulation and ADME/T analysis. The findings are consistent with experimental data, demonstrating that Schiff base 4Py exhibits greater inhibitory efficacy against S.aureus, while 3Py shows superior activity agains E. coli, compared to their respective HB complexes.

**Supplementary Information:**

The online version contains supplementary material available at 10.1038/s41598-025-09049-0.

## Introduction

Emergence of new infectious diseases and the development of drugs are the two common issues that attracted the attention of the researchers to investigate the new ways to combat them^1,2^. On the other hand, antimicrobial resistance in pathogens is also rapidly growing with multi-drug resistant (MDR) and showing resistance to many potential antibiotics. This situation poses a significant threat to public health due to inadequate options for treating MDR infections, accompanied by morbidity, mortality, and expensive treatments^3^. The MDR bacterial infections were caused to death to an estimation of about 5 million worldwide^4^ in 2019. This number may reach to 10 million annually by 2050 as per the projections which may impact the global economy with a potential loss of approximately $100 trillion, if effective measures are not taken^5^. To address this issue, the researchers are striving to invent the potential antimicrobial agents to combat MDR pathogens^6^.

A substituted aldehyde and a substituted aniline react with each other to form an imine, which is also known as Schiff base. They are also called as azomethines. The Schiff bases and their metal complexes are found to exhibit wide spectrum of biological activities^7–11^ viz., antibacterial, antifungal, antiseptic, antitumor, antioxidant, antiviral etc. Alamri et al. reported that the newly synthesised chitosan Schiff bases exhibited superior antimicrobial activity than the standard antibiotics like amoxicillin and tetracycline^13^. Novel thiazole-chloranils Schiff base derivatives synthesized by Sharma et al. showed an enhanced antibacterial and antifungal activity^14^. The physical, chemical and biological activities of Schiff base are reported to depend on the nature, size and number of the substituents^7^. Pyridine derivatives are reported to show wide spectrum of biological activities^18–20^.

The Hydrogen bonding (HB) interactions are strongest among the secondary forces of chemical interactions^21^ and are like tiny magnets between molecules. They gained much attention in recent years due to their ability to form the self-assembled^22^ structures, which influence the physical and chemical properties. Higher the electronegativity difference between hydrogen and an electronegative (EN) atom which involves in interactions, stronger will be the HB interactions. The nature of substituents i.e., electron-donating or electron-withdrawing, on either of the participating moieties also influence the strength of HB interactions and the other properties. The HB interactions are reported^27^ to play an important role in antimicrobial activity in therapeutic compounds by interfering with pathogens at the molecular level. Pyridine derivatives form strong HB interactions^28^ with other molecules, especially with hydroxyl group of the carboxylic acids. The 4-*n*-alkyloxybenzoic acids^32^ were exploited as proton donors in the formation of intermolecular HB with various Schiff base containing pyridine (proton acceptor) derivatives and realized the enhanced mesomorphism. The supramolecular structures^25^ formed through intermolecular HB interactions between complementary functional groups are witnessed^43^ for their vast applications in material science. The Schiff base compounds containing long chain carboxylic acids^44^ or alcohols^45^ or pyridine moiety^15^ are reported with potential antibacterial properties through their in vitro and in silico studies. In a study previously published by Marmer et al. , the authors reported the synthesis of a series of novel Schiff bases and evaluated their antimicrobial activity against six organisms: *Staphylococcus aureus* ATCC 25923, *Enterococcus faecalis* ATCC 29212, *Escherichia coli* ATCC 25922, *Pseudomonas aeruginosa* ATCC 27853, *Klebsiella pneumoniae* ATCC 13883, and *Acinetobacter haemolyticus* ATCC 19002, along with their antioxidant and urease activity. The study demonstrated that all synthesized compounds exhibited antimicrobial activity. It also included molecular docking studies of three Schiff bases with the urease enzyme from *Helicobacter pylori*. ADME/T analysis of the synthesized compounds was also carried out. While the study effectively explored the therapeutic potential of Schiff bases, it did not involve any structural modifications or derivative formations beyond the initial Schiff base synthesis. Moreover, molecular docking was performed only with *H. pylori* urease^15^.

However, a systematic study on the antibacterial activity of the HB complexes formed between Schiff base containing pyridine and 4-*n*-alkyloxybenzoic acids are not found in the literature. In comparison to the published work by Marmer et al. , the novelty of the current study lies in the evaluation of complex formation between the Schiff bases and 4-n-alkoxybenzoic acids (*n*OBA), along with a comprehensive investigation of the antimicrobial activity of the individual Schiff bases, *n*OBA derivatives, and their corresponding HB complexes against *E. coli* and *S. aureus*. Experimental analysis was validated by performing conventional molecular docking and molecular dynamics (MD) simulation using target proteins specific to *E. coli* and *S. aureus*, providing direct insight into the compounds’ interaction with the tested pathogens. In addition to conventional molecular docking of the individual ligands, we performed multiple ligand simultaneous docking to assess the potential synergistic interactions of the HB complexes with the mentioned bacterial targets. Furthermore, MD simulations were carried out for the Schiff bases and the HB complexes that exhibited significant antimicrobial activity, to gain insight into their stability and binding behaviour at the molecular level^15^. Therefore, an attempt has been made to understand the effect of the position of pyridyl nitrogen in the Schiff base and its HB interactions on antibacterial properties against Gram-positive and Gram-negative bacteria. in vitro antibacterial activities of all the synthesized HB complexes were carried out against *S. aureus* and *E. coli*, which were further validated by in silico molecular docking and MD simulation analysis to demonstrate the inhibitory action of key enzymes viz., GyraseB of *S. aureus*, and MurB of *E. coli.* ADME/T analysis were also carried out to study the drug-likeliness properties of the compounds.

## Experimental

### Materials and methods

The commercially available starting materials with high purity (> 99%) viz., 3-chloro-4-fluoroaniline, 4-pyridine carboxaldehyde, and 3-pyridine carboxaldehyde, 4*-n-*alkyloxybenzoic acids (*n*OBAs; ethyl, propyl and butyl) were purchased from Sigma-Aldrich. Acetic acid, Dimethylsulphoxide (DMSO) and Tetrahydrofuran (THF) were purchased from E. Merck, India Ltd. Muller Hinton Agar and streptomycin were obtained from Himedia. The chemicals and reagents were used as they receive from suppliers, without any further purification. Ethanol was procured from local distilleries and is double distilled. Aluminium sheets precoated with silica gel were used for thin layer chromatography (TLC), to monitor the progression / completion of reactions and to check the purity of the products. Shimadzu-8701 FTIR spectrometer was used for recording the FTIR spectra of the products. Bruker (Ascend, 400 MHz) Nuclear Magnetic Resonance (NMR) spectrometer was used to record ^1^H and ^13^C NMR spectra of the synthesized Schiff base compounds with tetramethyl silane (TMS) as an internal standard. Raman spectra were recorded using i-Raman^®^ Plus system with a 785 nm excitation wavelength. For antimicrobial assay studies, *Escherichia coli* (ATCC25922) and *Staphylococcus aureus* (ATCC29737) strains were collected from the culture collection center, Department of Microbiology, Kasturba Medical College, Manipal, India. The computational studies with a geometry optimization and density functional theory (DFT) were carried out using def2-SVP basis set, considering a total charge of zero and a singlet spin multiplicity (multiplicity = 1).

### Synthesis and characterization of pyridine containing schiff bases

**Scheme 1:**
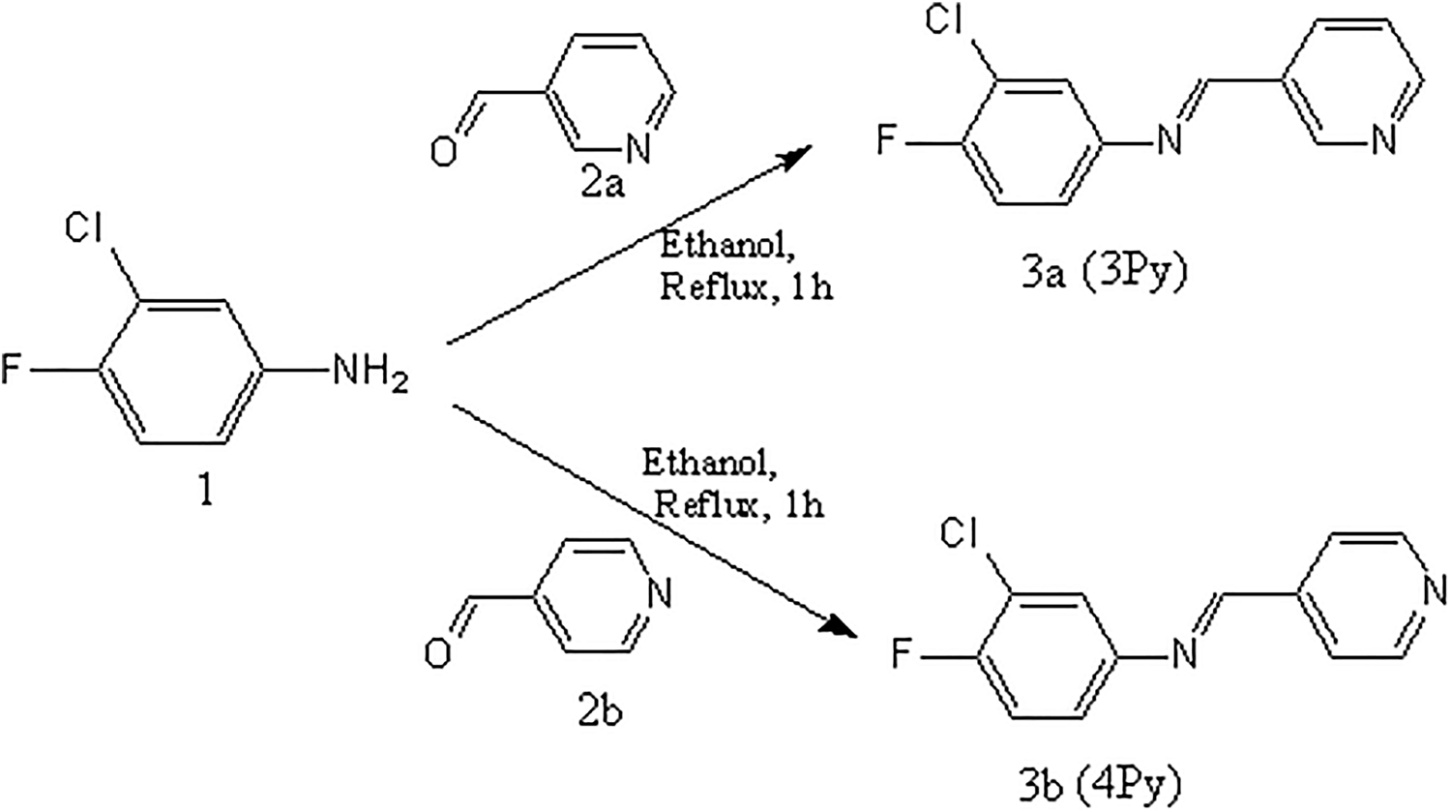
Synthetic route for the preparation of Schiff bases.

The Schiff base containing pyridine moieties viz. 3-chloro-4-fluoro-N-((pyridin-3-yl)methylene)benzenamine (3Py) and 3-chloro-4-fluoro-N-((pyridin-4-yl)methylene)benzenamine (4Py) were prepared by condensing the corresponding aldehydes and anilines in 1:1 molar ratio as reported^15^ earlier. To an ethanolic solution of 3-chloro-4-fluoroaniline (1a), an equimolar quantity of 3-pyridinecarboxaldehyde (2a) and 3 drops of glacial acetic acid were added. The contents were refluxed for 1 h. followed by the distillation of the solvent, yield a white crystalline product, Schiff base (3Py, **3a**). The product was recrystallised thrice. Similarly, 3-chloro-4-fluoroanilne (1a) and 4-pyridinecarboxaldehyde (2b) were condensed to yield the product, 4Py (**3b**). The synthetic route for the preparation of 3Py and 4Py are depicted in Scheme 1.

The compounds 3Py (3a) and 4Py (3b) were characterized by FTIR, ^1^H and ^13^C NMR spectroscopy techniques. The respective spectra are given in supplementary information file (fig. S1 to fig. S6). The melting points of 3Py and 4Py were determined by open capillary method and were found to be 93.7^0^C and 81.2^0^C respectively. The melting points and the spectral data of the products were in good agreement with the similar Schiff bases reported^15^.

### Preparation and characterization of HB complexes

**Fig. 1:**
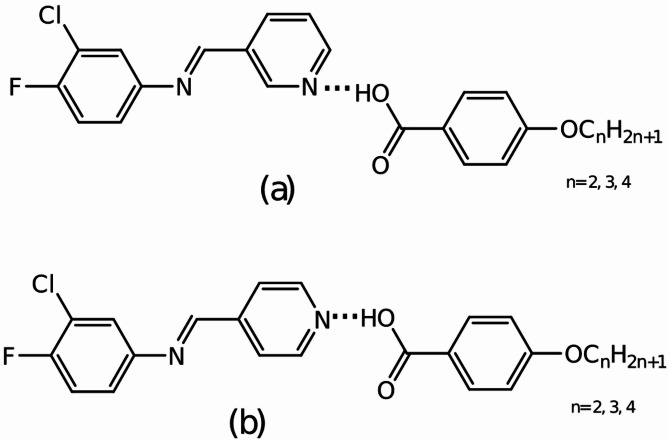
The general molecular structure of the HB complexes (a) 3Py:*n*OBA and (b) 4Py:*n*OBA.

The HB complexes of the Schiff base with pyridine moieties were prepared by treating 1:1 molar ratio THF solutions of 3Py and 4-*n*-alkyloxybenzoic acid (*n*OBA; ethyl, propyl and butyl). The solution was refluxed for 2 h. followed by distillation of the solvent, yielding the HB complexes (3Py:*n*OBA). Similarly, the HB complexes, 4Py:*n*OBA were prepared. The general molecular structure of the complexes with possible HB interactions are presented in Fig. 1.

### Methodology for *in vitro* antibacterial activity by Kirby-Bauer’s disc diffusion assay

The antibacterial activity of the pristine compounds viz., 3Py, 4Py and 4-*n*-alkyloxybenzoic acids (2OBA, 3OBA, and 4OBA), and their HB complexes, were assessed against *S. aureus* and *E. coli.* The evaluation was conducted using a standard paper disc diffusion method, as described by Bauer and Kirby, and in accordance with guidelines from the American society for Microbiology^47^. The method involves the preparation of the discs followed by inoculation, incubation, measurement reproducibility and statistical analysis were described below.

### Preparation of the discs

The antibiotic paper discs were prepared by using Whatman filter paper No. 1 and their discs were sterilized using autoclave. The test compounds viz., 3Py, 4Py, 2OBA, 3OBA, 4OBA and their HB complexes, 3Py:2OBA, 3Py:3OBA, 3Py:4OBA, 4Py:2OBA, 4Py:3OBA and 4Py:4OBA were accurately weighed and dissolved in DMSO. The solvent, DMSO was chosen as all the compounds in the present studies were soluble in it and it has no antibacterial activity (i.e., negative control). Each disc was impregnated with 100 µg of the test compound and dried in a hot air oven in sterile conditions.

### Inoculation and incubation

The prepared discs were placed on Mueller-Hinton Agar (MHA) plates that had been evenly inoculated with fresh bacterial suspensions adjusted to a 0.5 McFarland standard using a bioSan DEN-1B densitometer. The plates were incubated at 37 °C for 24 h.

### Quality controls and measurement reproducibility

Standard streptomycin (10 µg) disc was used as a positive control, while DMSO-impregnated discs acted as negative controls. After incubation, zones of inhibition were measured in millimeters to assess antibacterial activity. To ensure reliability and reproducibility, all experiments were conducted in triplicates. The performance of the HB complexes was compared with their individual proton donors and proton acceptors.

### Statistical analysis

All the tests were conducted in triplicates and values are represented in the form of *n* ± SD, where ‘*n*’ is the average of the 3 trails. Statistical analysis of the test was done as per the two tailed student’s t-test and plotted. Mean comparisons were conducted with the least significant difference (LSD) (*P* = 0.05) test.

### Computational studies

Computational studies have been carried out for the compounds, which involve geometry optimization and density functional theory calculations followed by the molecular docking and dynamics studies. The methodology for the same is described below.

### Geometry optimization and density functional theory (DFT) calculations

Schiff bases, i.e., 3Py and 4Py were used to perform computational analysis. The input file for the DFT calculations was generated using Avogadro, and DFT calculations were performed using ORCA (version 6.0.1)^48^. Geometry optimization was performed using the Universal Force Field (UFF) in four steps, employing the steepest descent algorithm. The resulting energy-minimized geometry was then used to generate the ORCA input file. A geometry optimization calculation was subsequently carried out using DFT with the def2-SVP basis set, considering a total charge of zero and a singlet spin multiplicity (multiplicity = 1). The ORCA input file was generated in Cartesian coordinate format to facilitate DFT analysis. The molecular descriptor, such as the HOMO-LUMO gap, was also computed at the same theoretical level.

### Molecular docking and dynamics investigations

The molecular docking and dynamics studies involve the protein and ligand preparation followed by the simulation studies as described below.

#### Protein and ligand preparation

The standard antibiotic streptomycin, along with the synthesized Schiff bases viz., 3Py and 4Py and their respective HB complexes with 2OBA (i.e., 3Py:2OBA and 4Py:2OBA), were used as ligands. The structure of streptomycin was obtained from the PubChem database (https://pubchem.ncbi.nlm.nih.gov/). The structures of Schiff bases were drawn using the 2D sketcher feature in Schrödinger Maestro version 2024-2. The saved pdb files were converted to pdbqt format using Open Babel.

For the target proteins, MurB, a protein involved in cell wall synthesis of *E. coli*, and *S. aureus* GyraseB enzyme were considered for analysis of the synthesized ligands within the targets. The three-dimensional coordinates of *E. coli* MurB (PDB id: 1MBT)^49^ and *S. aureus* GyraseB (PDB id: 4URO)^50^ were downloaded from the Protein Data Bank (https://www.rcsb.org/).

Before docking, the standard antibiotic and the synthesized compounds, the target proteins were prepared using AutoDock Tools (ADT)^51^. The target proteins were made free from the co-crystallized ligands. Subsequently, the apoproteins were prepared by eliminating the water molecules, adding polar hydrogen, and adding Kollman charges, and the prepared structures were saved in pdbqt format.

#### Molecular Docking

Molecular docking was performed via AutoDock Vina^52,53^. Both single ligand docking and multi-ligand simultaneous docking were employed for the study. Single ligand docking was performed for Schiff bases complexes, 3Py with *E. coli* MurB and 4Py with *S. aureus* GyraseB. In contrast, multi-ligand simultaneous docking was employed for their corresponding HB complexes with 2OBA. For docking the ligands within the active site of *E. coli* MurB, the grid box over the active site of the protein was created with dimensions 16 × 16 × 16, with their positioning along the X, Y, and Z axes at 6.929, 34.632, and 22.616, respectively. Similarly, the active site grid box dimension for *S. aureus* GyraseB was 30 × 30 × 30 with the grid centered at -6.8, -6.47, and − 13.16 along the X, Y and Z axes respectively. Grid boxes for both proteins were generated, keeping the spacing at 1 Å. The exhaustiveness for *E. coli* MurB was set as 20 while that for *S. aureus* GyraseB was set to 24. Docking scores of the synthesized compounds and their HB complexes were compared with the standard antibiotic streptomycin. Molecular interactions of the ligands post docking were analyzed using Ligplot+^54^.

#### MD simulations

MD simulations were conducted for 100 ns with a simulation pH 7.4 on Schiff base complexes, 3Py with *E. coli* MurB and 4Py with *S. aureus* GyraseB, and their respective HB complexes with 2OBA using the Desmond module of the Schrödinger suite (version 2024-2)^55^. The complex systems were constructed employing the SPC water model, with a solvent box extending 10Å × 10Å × 10Å from the protein surface. Simulations were performed under an NPT ensemble at 310 K, with trajectory data recorded at 100 ps intervals.

### Analysis of drug-likeness properties

The compounds exerting better antimicrobial activity with *E. coli* and *S. aureus* were analyzed for their drug-likeness properties. ADMET of 2OBA was also analyzed since it is involved in HB complex with the Schiff bases. SwissADME webserver was used for the analysis of Lipinski’s rule of five (RO5), Ghose, Veber, Egan, and Muegge rules (http://www.swissadme.ch/)^56^.

## Results and discussion

### IR spectra

In the FTIR spectrum of the Schiff base (3Py), the absorption bands above 3100 cm^− 1^ are absent, which confirms the involvement of -NH_2_ group of 3-chloro-4-fluoroaniline in Schiff base formation. The absorption band corresponding to C = O stretching of pyridine carboxaldehyde in the range 1715–1695 cm^− 1^ is also absent, which indicates the condensation between the aniline and carboxaldehyde. The C–H stretching of aromatic ring and pyridine are noticed at around 3000 cm^− 1^. A peak at 1625 cm^− 1^ infers the C = N stretching of Schiff base while the C = N stretching of pyridine moiety is observed by a peak at 1570 cm^− 1^. The FTIR, ^1^H and ^13^C NMR spectra of other Schiff base, 4Py are given in supporting information as fig. S4 to fig. S6.

**Fig. 2:**
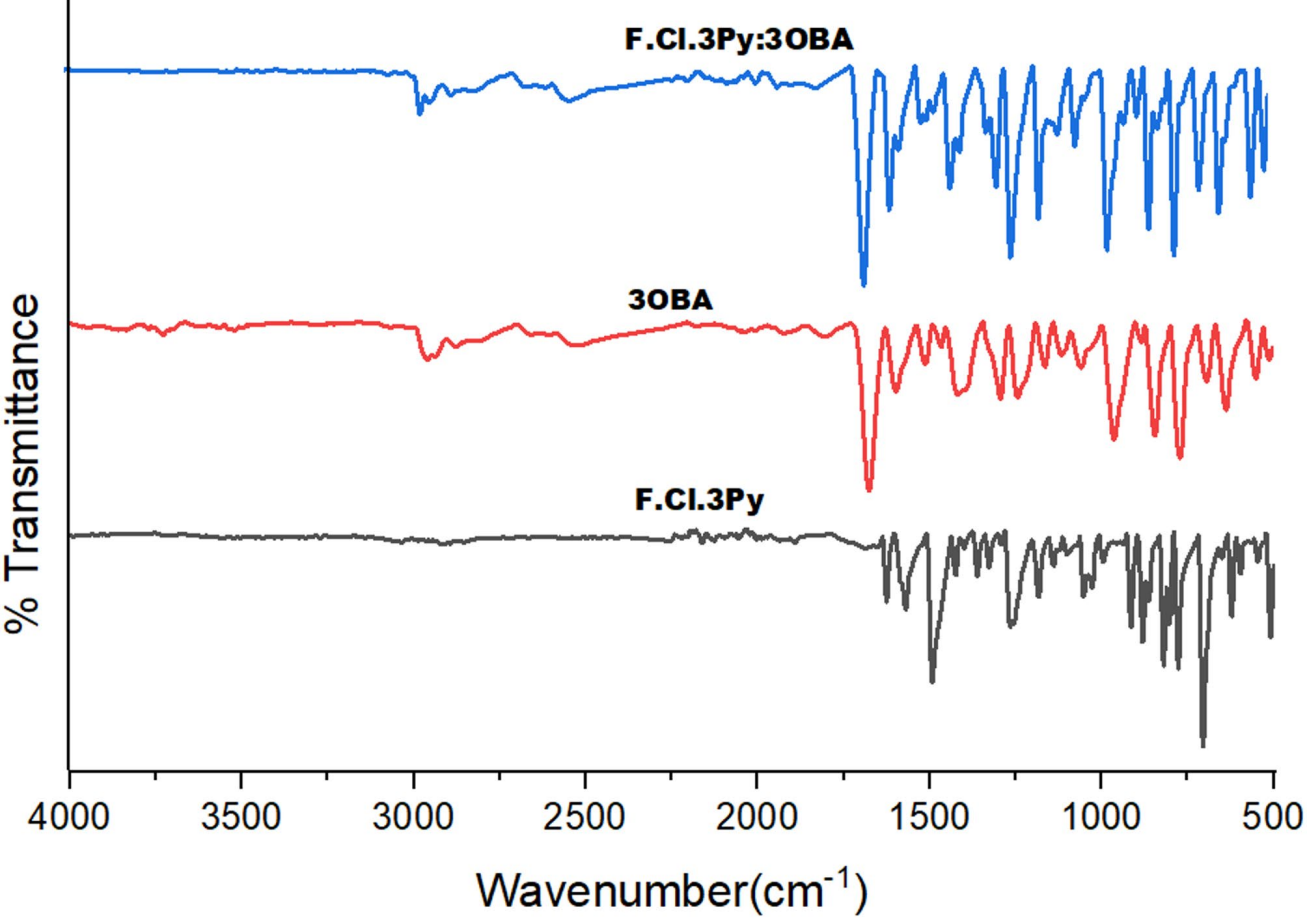
FTIR spectra of 3Py, 3OBA and 3Py:3OBA complex.

All the HB complexes viz., 3Py:*n*OBA and 4Py:*n*OBA (*n* = 2, 3 and 4) are characterized by FTIR and as a representative member, the FTIR spectrum of 3Py:3OBA is given in Fig. 2  along with its constituents. When compared the FTIR spectrum of 3Py:3OBA with 3Py (proton acceptor) and 3OBA (proton donor), new absorption bands at 2965 and 2537 cm^− 1^ were observed which confirms^29^ the presence of non-ionized intermolecular HB interactions between carboxylic acid group and pyridine nitrogen i.e., lone electron pair of pyridine nitrogen and hydroxylic acid group. The FTIR spectra of 4Py, 3OBA and 4Py:3OBA complexes are given in supplementary information as fig. S7 as a representative of the series.

### Raman spectra

The Raman spectra of the Schiff bases (3Py and 4Py), 4-butyloxybenzoic acid (4OBA), as a representative member, and their respective HB complexes viz., 3Py:4OBA and 4Py:4OBA were analysed to identify characteristic vibrational modes. In 4OBA, the C–H stretching vibrations are typically observed in the range of 3000–3100 cm^−1^, appearing as faint peaks at 3083.1 cm^−1^ (Supplementary information fig. S8). The characteristic C = O stretching vibrations are detected in the range of 1600–1850 cm^−1^, while the C–O vibrational frequencies appear between 1200 and 1300 cm^−1^^57^. Additionally, C–H bending and stretching vibrations are observed within the 1200–1650 cm^−1^ region, which is consistent in both 4OBA and the Schiff base complexes. A comparative analysis of the Raman spectra of the individual compounds and their respective HB complexes reveals that the HB complexes exhibit peaks corresponding to both parent molecules, indicating the preservation of key functional groups.

### *In vitro* antibacterial activity studies

**Fig. 3:**
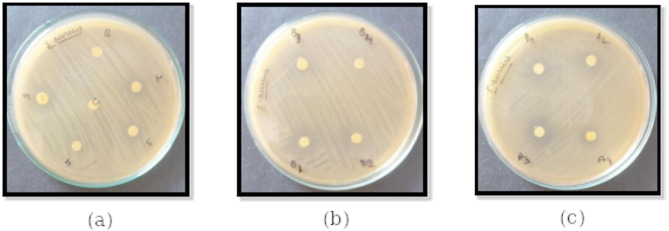
Antibacterial activity of (**a**) 4-*n*-alkyloxybenzoic acids (**b**) 3Py:*n*OBA (*n* = 2, 3, and 4), and (**c**) 4Py:*n*OBA (*n* = 2, 3, and 4) against *S. aureus*.

The antibacterial activities of pristine compounds viz., 3Py, 4Py, 4-*n*-alkyloxybenzoic acids (2OBA, 3OBA, and 4OBA), and their HB complexes were studied against *S. aureus* and *E. coli.* The results of antibacterial activity (inhibition zone of discs) against *S. aureus* are given in Fig. 3. The letters B, C, S, 2, 3, and 4 in Fig. 3(a) denotes blank, control, Streptomycin (standard antibiotic), 2OBA, 3OBA, and 4OBA, respectively. The letters viz., B_1_, B_2_, B_3_ and B_4_ in Fig. 3(b) denotes 3Py, 3Py:2OBA, 3Py:3OBA and 3Py:4OBA respectively. In Fig. 3(c), A_1_, A_2_, A_3_ and A_4_ denotes 4Py, 4Py:2OBA, 4Py:3OBA and 4Py:4OBA respectively.

**Fig. 4:**
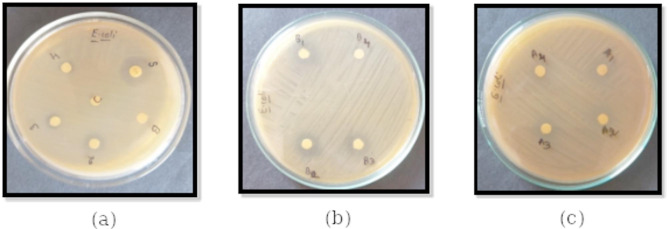
Antibacterial activity of (**a**) 4-n-alkyloxybenzoic acids, (**b**) 3Py:*n*OBA (*n* = 2, 3, and 4) and (**c**) 4Py:*n*OBA (*n* = 2, 3, and 4) against *E. coli*.

The results of antibacterial activity (inhibition zone of discs) against *E. coli* are given in Fig. 4. In Fig. 4(a), B, C, S, 2, 3, and 4 denote blank, control, streptomycin (standard antibiotic), 2OBA, 3OBA, and 4OBA, respectively. The letters viz., B_1_, B_2_, B_3_ and B_4_ in Fig. 4(b) denotes 3Py, 3Py:2OBA, 3Py:3OBA and 3Py:4OBA respectively. In Fig. 4(c); A_1_, A_2_, A_3_ and A_4_ denotes 4Py, 4Py:2OBA,.4Py:3OBA and 4Py:4OBA respectively.

**Fig. 5:**
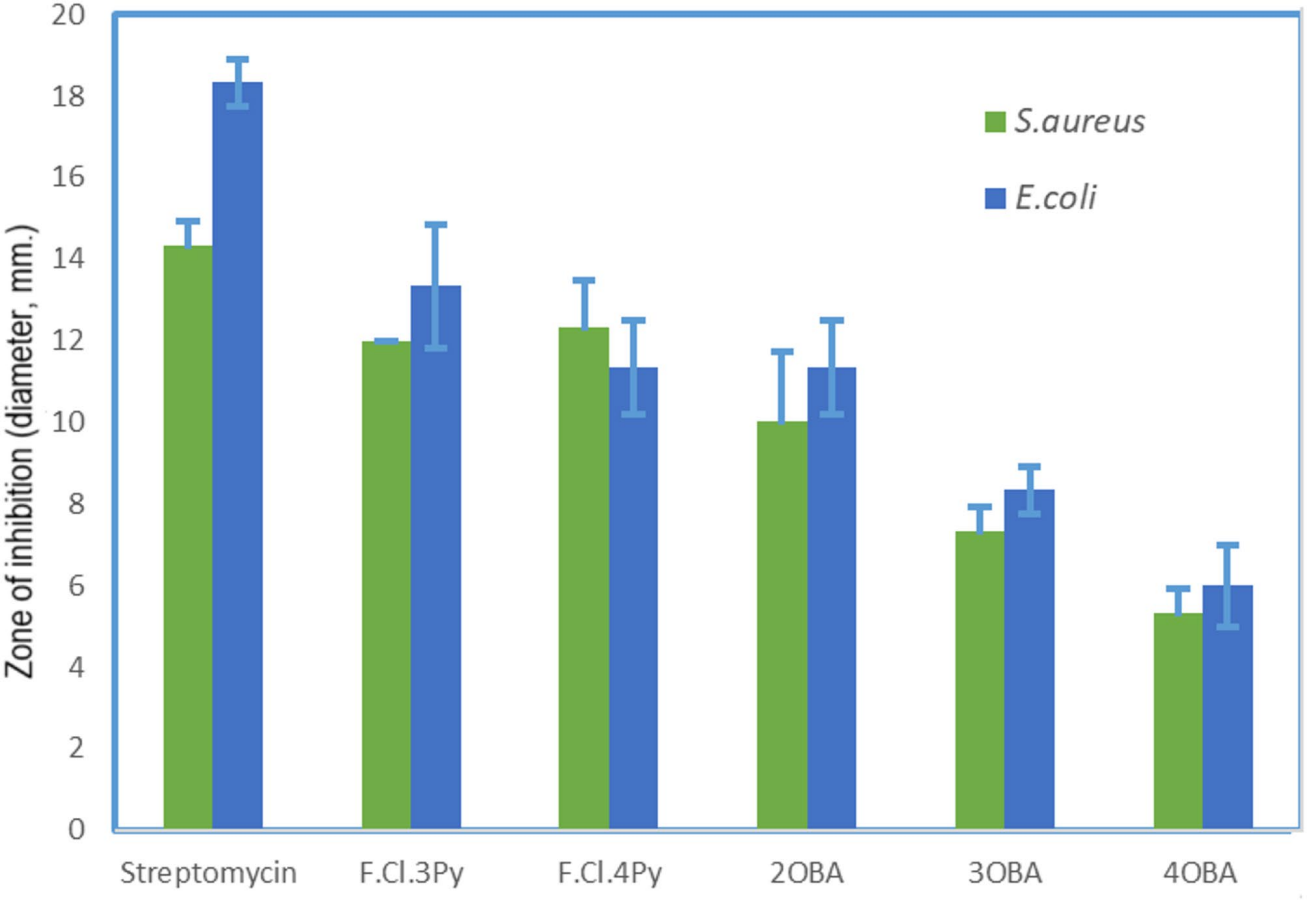
Comparision of antibacterial activities of streptomycin (standard antibiotic), Schiff bases 3Py (F.Cl.3Py) and 4Py (F.Cl.4Py) and 4-*n*-alkyloxybenzoic acids against *S. aureus* and *E. coli.*

The inhibition zone values (mm) are given in the Table 1 and are analysed statistically using student’s t-test with a significance level of 0.05. The inhibition zones (diameter in mm) of *S. aureus* and *E. coli* observed against the prestine compounds are compared and is given in Fig. 5. It is noticed that the Schiff bases with pyridine moiety i.e., 3Py and 4Py showed appreciable activity against both the *S. aureus* and *E. coli* when compared with 2OBA, 3OBA and 4OBA. The pyridine derivatives are reported^58^ with potential biological activities against various bacterial strains^46^. However, the exact mechanism of biological activity is not discussed, but the probable mechanism of antimicrobial action is due to the inhibitory nature of various enzymes like Thymidylate Kinase, GlcN-6-P Synthase, DNA Gyrase^60^ etc.

**Table 1:** Antibacterial activity of 3Py, 4Py, 4-*n*-alkyloxybenzoic acids and their HB complexes.

Compounds	Zone of inhibition (mm)	
	*S. aureus*	*E. coli*
Streptomycin (Positive control)	14.33 ± 0.57	18.33 + 0.57
DMSO (Negative Control)	0.00	0.00
3Py	12.00 ± 0.00	13.33 ± 1.52
4Py	12.33 ± 1.15	11.33 ± 1.15
2OBA	10.00 ± 1.73	11.33 ± 1.15
3OBA	7.33 ± 0.57	8.33 ± 0.57
4OBA	5.33 ± 0.57	6.00 ± 1
3Py:2OBA	10.00 ± 1.00	10.66 ± 0.57
3Py:3OBA	8.66 ± 0.57	8.66 ± 0.57
3Py:4OBA	5.00 ± 00	5.33 ± 0.57
4Py:2OBA	11.33 ± 1.15	10.66 ± 0.57
4Py:3OBA	10.66 ± 2.08	8.66 ± 0.57
4Py:4OBA	8.33 ± 1.52	5.33 ± 0.57

From Fig. 5, it is very clear that the 4Py (denoted as F.Cl.4Py in Fig. 5) showed better antibacterial activity against *S. aureus* than the 3Py (denoted as F.Cl.3Py in Fig. 5), which is in agreement with the reported data^15^. Interestingly, it is observed that the 3Py showed better antibacterial activity against *E. coli* than the 4Py. In both the cases i.e., 3Py and 4Py, the position and nature of the halogen substituents on Schiff base are the same and the molecules are differed only in the relative position of pyridyl nitrogen. These observations infer that the position of pyridyl nitrogen influence the antibacterial activity. The effect of chain length of 4-*n*-alkyloxybenzoic acids on antimicrobial activity is given in Fig. 5. It is observed that the *n*OBAs in the present study showed better antibacterial activity with *E. coli* than *S. aureus*. It is also noticed that the antimicrobial activity decreased with an increase of alkyl chain length for both *S. aureus* and *E. coli.* This is in concurrence with the observations of Wood et al. on the antimicrobial activity of (E)-3-alken-2-ones with different chain lengths.

**Fig. 6:**
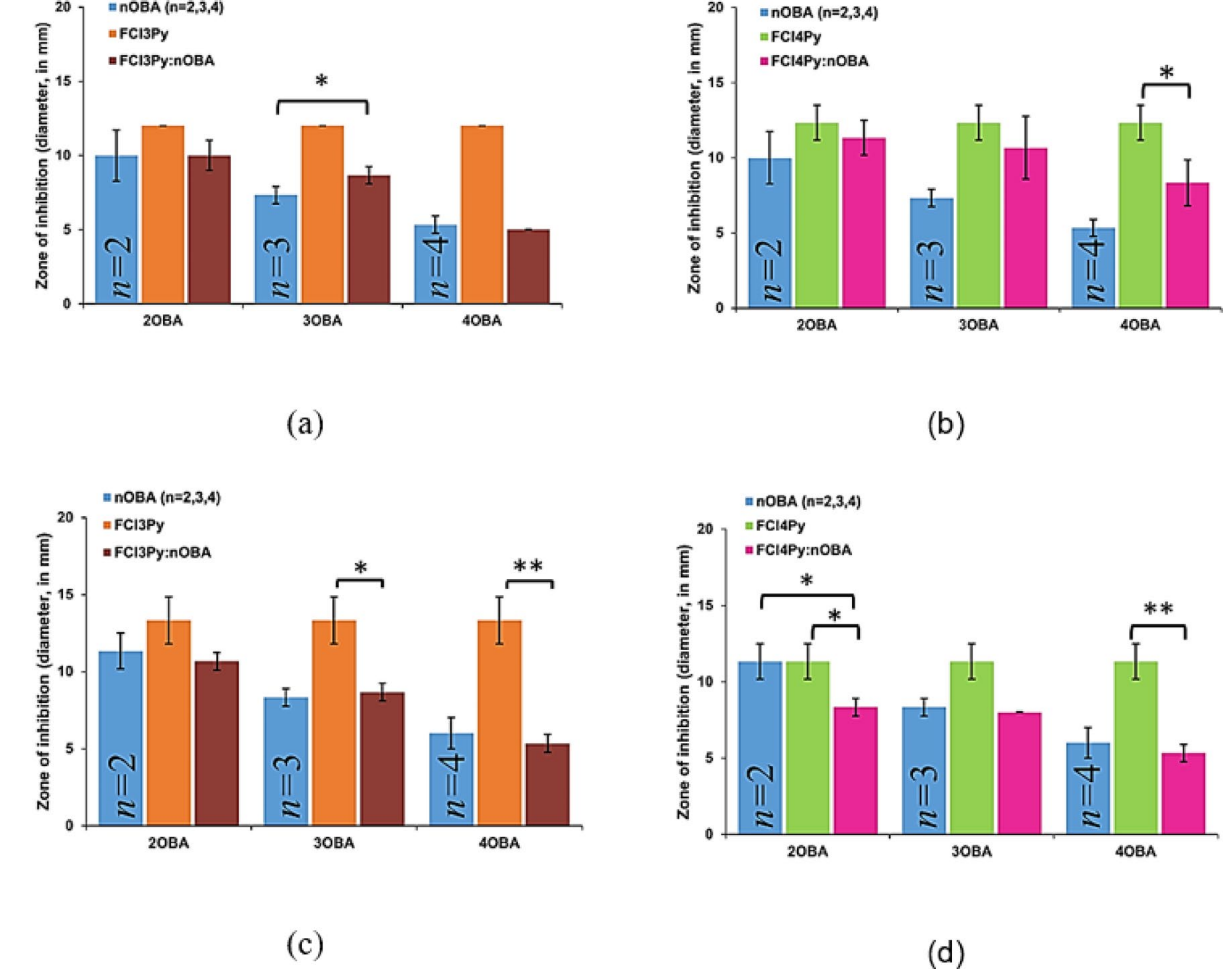
Comparison of antibacterial activities of (**a**) *n*OBA, 3Py and 3Py:*n*OBAs against *S. aureus;* (**b**) *n*OBA, 4Py and.4Py:*n*OBAs against *S. aureus*; (**c**) *n*OBA, 3Py and 3Py:*n*OBAs against *E. coli* (**d**) *n*OBA, 4Py and.4Py:*n*OBAs against *E. coli.*

The influence of HB interactions between 3Py or 4Py (proton acceptors) and *n*OBA (proton donors) on the antibacterial properties against *S. aureus* and *E. coli* have been studied and compared with the respective entities and are given in Fig. 6. It is observed that the 3Py and 4Py showed higher activity than the *n*OBA and the corresponding HB complexes viz., 3Py:*n*OBA or 4Py:*n*OBA as shown in Fig. 6 against *S. aureus* and *E. coli*. The statistical analysis also supports the significant decrease in the antibacterial activity of the HB complexes when compared with the 3Py and 4Py.

Interestingly, it is noticed that the activity of HB complexes is higher than the corresponding *n*OBAs except 3Py:4OBA against *S. aureus* as shown in Fig. 6(a) and 6(b). This is also aided by a statistical test and the activity of 3Py:3OBA is found to increase significantly compared to 3OBA. Conversely, the activities of HB complexes are lower than the corresponding *n*OBAs against *E. coli* as shown in Fig. 6 (c) and 6(d). The results obtained are further validated by computational studies.

###  Computational studies

*E. coli* is regarded as one of the organisms responsible for antimicrobial resistance. The bacterial cell wall peptidoglycan layer is one of the crucial targets for antibiotics since it is necessary to maintain the bacterial osmotic balance. The process of peptidoglycan synthesis is catalyzed by two enzymes- MurA and MurB, in a two-stage process^49^ MurA initiates this process by transferring an enolpyruvate group from phosphoenolpyruvate to UDP-N-acetylglucosamine (UDP-GlcNAc), forming enolpyruvyl-UDP-N-acetylglucosamine (EP-UDP-GlcNAc) and releasing inorganic phosphate. MurB then terminates the process by converting the enolpyruvyl group into a lactyl ether with the help of one molecule of NADPH, resulting in the formation of the muramyl sugar^49^. The lactyl ether functions as a crucial linker between the peptide and glycan components of the UDP-muramyl peptide. Given the absence of analogous reactions in eukaryotic metabolic pathways, MurB presents a highly selective and promising target for antibacterial drug development^63^.

Moreover, the rise of drug-resistant *S. aureus* has made first-line antibiotics ineffective against infections, increasing the mortality and morbidity rates globally^50,64,65^, The organism adapts to antibiotics through both natural and acquired resistance mechanisms, allowing it to survive drugs like fluoroquinolones, vancomycin, and carbapenems, making it necessary to develop new, safer, and equally effective antibiotics. Since the proteins involved in bacterial DNA replication are different from those in humans but are highly conserved among bacterial species, they present an opportunity to discover broad-spectrum antibiotics with minimal side effects. One of the most promising targets is DNA Gyrase, a crucial enzyme for bacterial DNA replication. It unwinds the DNA ahead of the replication fork and prevents breaks in the DNA strand by introducing negative supercoils using ATP. DNA gyrase is a heterotetrameric enzyme with two subunits: GyraseA and GyraseB^64^. GyraseA is responsible for cutting and rejoining DNA strands, while GyraseB powers the process by breaking down ATP to generate supercoiling. Targeting DNA gyrase represents a promising strategy for the development of highly effective new antibiotics^64^. Therefore, in silico analysis considered two important targets, *E. coli* MurB and *S. aureus* GyraseB, for evaluating the interaction of the synthesized compounds within their active sites.

#### **Frontier molecular orbital (FMO) analysis**

The Highest Occupied Molecular Orbital (HOMO) and Lowest Unoccupied Molecular Orbital (LUMO), collectively termed Frontier Molecular Orbitals (FMO), govern molecular reactivity and interaction potential. HOMO acts as an electron donor, while LUMO serves as an electron acceptor, playing a key role in drug-target interactions. FMO analysis provides insights into binding affinity and molecular interaction mechanisms. Key molecular descriptors derived from FMO include ionization potential, electron affinity, electronegativity, electrophilicity, hardness, and softness, which define electron exchange behavior, molecular stability, and reactivity. These parameters are essential for understanding drug behavior and optimizing pharmacological applications^67^. The FMO analysis and key molecular descriptors of the Schiff bases analyzed in our study are summarized in supplementary information Table S1.

**Fig. 7:**
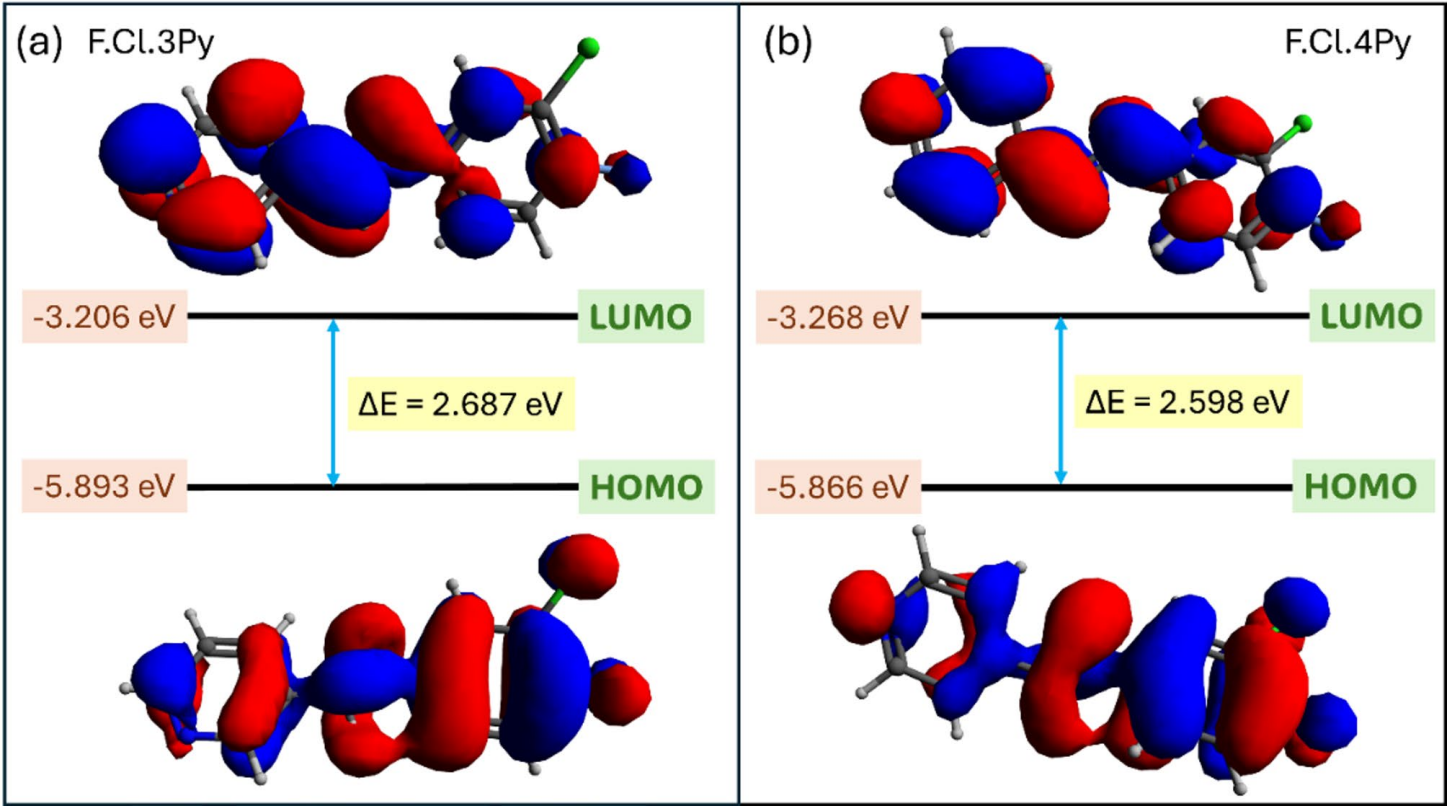
Frontier molecular orbitals of (**a**) 3Py (denoted as F.Cl.3Py in fig.) and (**b**) 4Py (denoted as F.Cl.4Py in fig.) computed by DFT. The HOMO-LUMO energy gap is represented as ∆E.

The FMOs of 3Py and 4Py were computed using DFT calculations, and their energy gaps are represented in Fig. 7. The FMO analysis of the Schiff bases 3Py and 4Py reports the HOMO-LUMO energy gap (ΔE) was calculated as 2.687 eV (Fig. 7a) and 2.598 eV (Fig. 7b), respectively. Both values are comparable, and the energy gap value is slightly lower for 3Py. Recent study by Kaavin et. al. indicates that the molecules with an FMO energy gap below 2.5 eV exhibit a high propensity for binding to specific protein targets^67^. Although the FMO analysis for the 3Py and 4Py (Schiff bases) is comparable to and slightly exceeds the reported threshold values, additional parameters must be considered to comprehensively assess the compound’s potential for promising interactions with the selected protein targets. Therefore, molecular docking, MD simulation, and ADME/T analysis were further performed to identify the potential of the compounds as antimicrobial agents.

#### **Molecular docking**

**Fig. 8:**
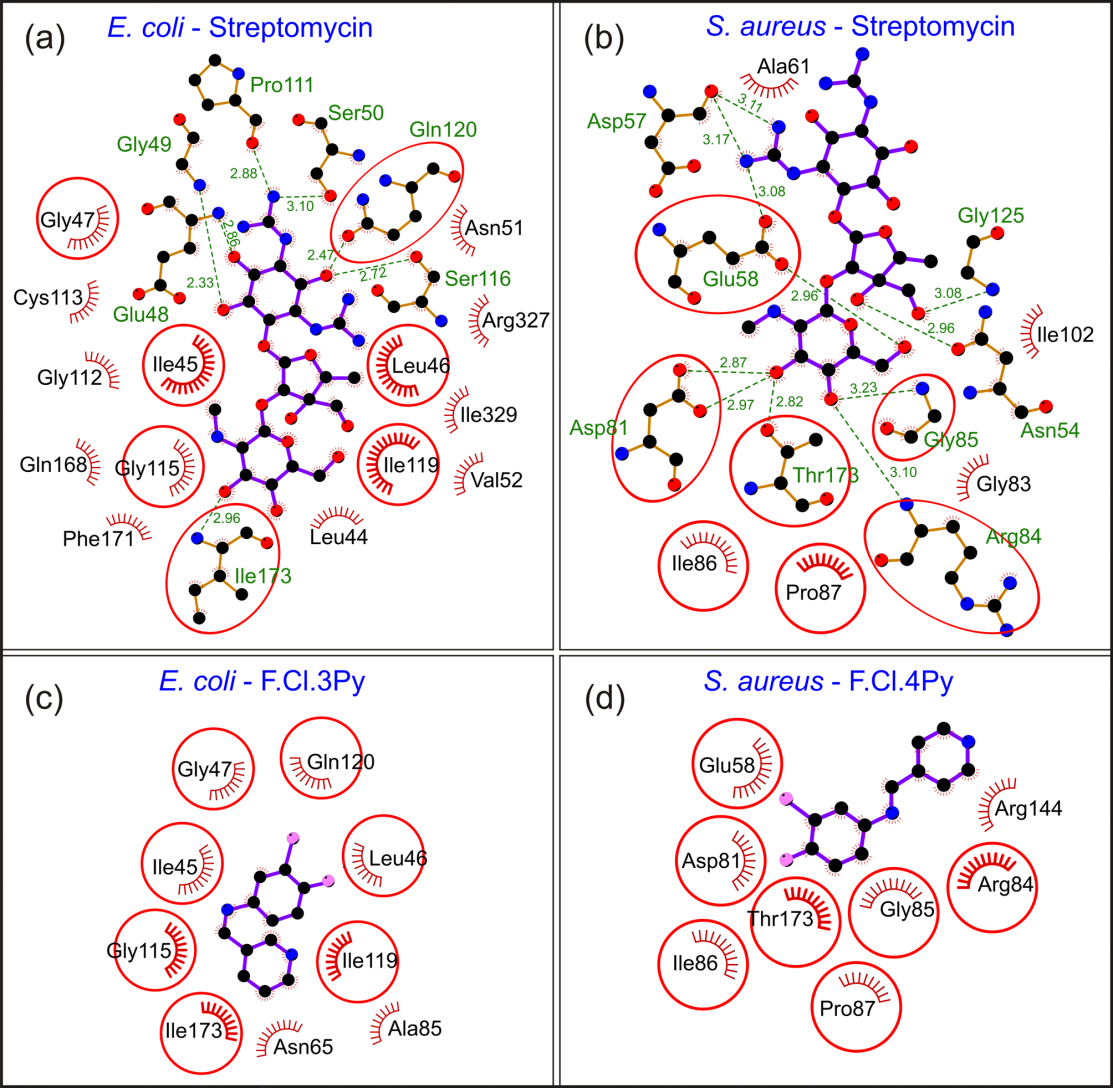
Molecular interaction between the standard antibiotic and Schiff bases in the catalytic site of *E. coli* MurB and *S. aureus* GyraseB. Interaction of streptomycin with (**a**) *E. coli* MurB and (**b**) *S. aureus* GyraseB, (**c**) *E. coli* MurB with 3Py (denoted as F.Cl.3Py in fig) and (**d**) *S. aureus* GyraseB with 4Py (denoted as F.Cl.4Py in fig).

Molecular docking is a computational method in drug discovery that predicts the optimal interaction between a protein and a ligand while evaluating their binding energy, aiding in the identification of potential drug candidates by analysing their binding efficiency and stability^68^. In the current study, the binding energies of the synthesized Schiff bases, 3Py and 4Py and their respective HB complexes with 2OBA were compared with the standard antibiotic streptomycin within the catalytic sites of *E. coli* MurB and *S. aureus* GyraseB, respectively. These compound complexes were selected as the representatives of the synthesized HB complexes to demonstrate* in silico* interactions. The binding energies and the molecular interactions of *E. coli* and 3Py and *S. aureus* and 4Py and the HB complexes are summarized in supplementary information Table 2. In the catalytic site of *E. coli* MurB, streptomycin obtained a binding energy of -4.44 kcal/mol while 3Py had a binding energy better than streptomycin which was obtained as -7.02 kcal/mol. For docking the HB complex of 3Py and 2OBA, multi-ligand simultaneous docking was employed, and the score obtained was − 10.85 kcal/mol (supplementary information Table S2). Figure 8 represents the molecular interaction of streptomycin with *E. coli* Mur B (Fig. 8a) and *S. aureus* GyraseB (Fig. 8b), interaction of *E. coli* MurB with 3Py (Fig. 8c) and *S. aureus* GyraseB with 4Py (Fig. 8d). Streptomycin formed interactions with the catalytic site residues of MurB via H-bonds and non-bonded interactions (Fig. 8a). Glu48, Gly49, Ser50, Pro111, Ser116, Gln120, and Ile173 contributed to H-bond formation. Leu44, Ile45, Leu46, Gly47, Asn51, Val52, Gly112, Cys113, Ile119, Gln168, Phe171, Arg327, and Ile329 were associated with non-bonded interactions (supplementary information Table S2). 3Py only interacts through non-bonded interactions via the residues Ile45, Leu46, Gly47, Asn65, Ala85, Gly115, Ile119, Gln120, and Ile173 (Fig. 8c). Except for Asn65 and Ala85, other residues are common among streptomycin and 3Py in the catalytic site of *E. coli* MurB. However, Gln120 and Ile173 form H-bonds with streptomycin while these residues contributed for non-bonded interactions for 3Py (Fig. 8a and c). These results support the experimental data discussed in “in vitro antibacterial activity studies”.

Within the catalytic site of *S. aureus* GyraseB, streptomycin showed a binding energy of -6.82 kcal/mol while 4Py showed a binding energy of -6.20 kcal/mol. The binding energies of both the compounds are comparable within the active site of GyraseB. This may be due to the smaller molecular weight of 4Py relative to streptomycin. Multi ligand simultaneous docking of 4Py and 2OBA obtained a binding energy of -8.66 kcal/mol. With respect to the molecular interactions, streptomycin showed H-bonds and non-bonded interactions (Fig. 8b) while 4Py participated only in non-bonded interactions. Asn54, Asp57, Glu58, Asp81, Arg84, Gly85, Gly125, and Thr173 are associated with H-bonds with streptomycin. Moreover, Ala61, Gly83, Ile86, Pro87, and Ile102 are involved in non-bonded interactions (Fig. 8b). 4Py is involved in non-bonded interactions in the catalytic site of *S. aureus* GyraseB via Glu58, Asp81, Arg84, Gly85, Pro87, Arg144, and Thr173 (Fig. 8d).

Residues involved in HB interactions are marked in green, while those participating in non-bonded interactions are indicated in red colour. Common interacting residues between streptomycin and Schiff bases with the respective bacterial targets are enclosed within red circles.

####  MD simulation analysis

MD simulations were performed for the compounds exhibiting significant antimicrobial activity in vitro. A 100 ns simulation trajectory analysis was conducted for the target protein complexes with Schiff bases i.e., *E. coli* MurB with 3Py and 3Py:2OBA and *S. aureus* GyraseB with 4Py and 4Py:2OBA.

**Fig. 9:**
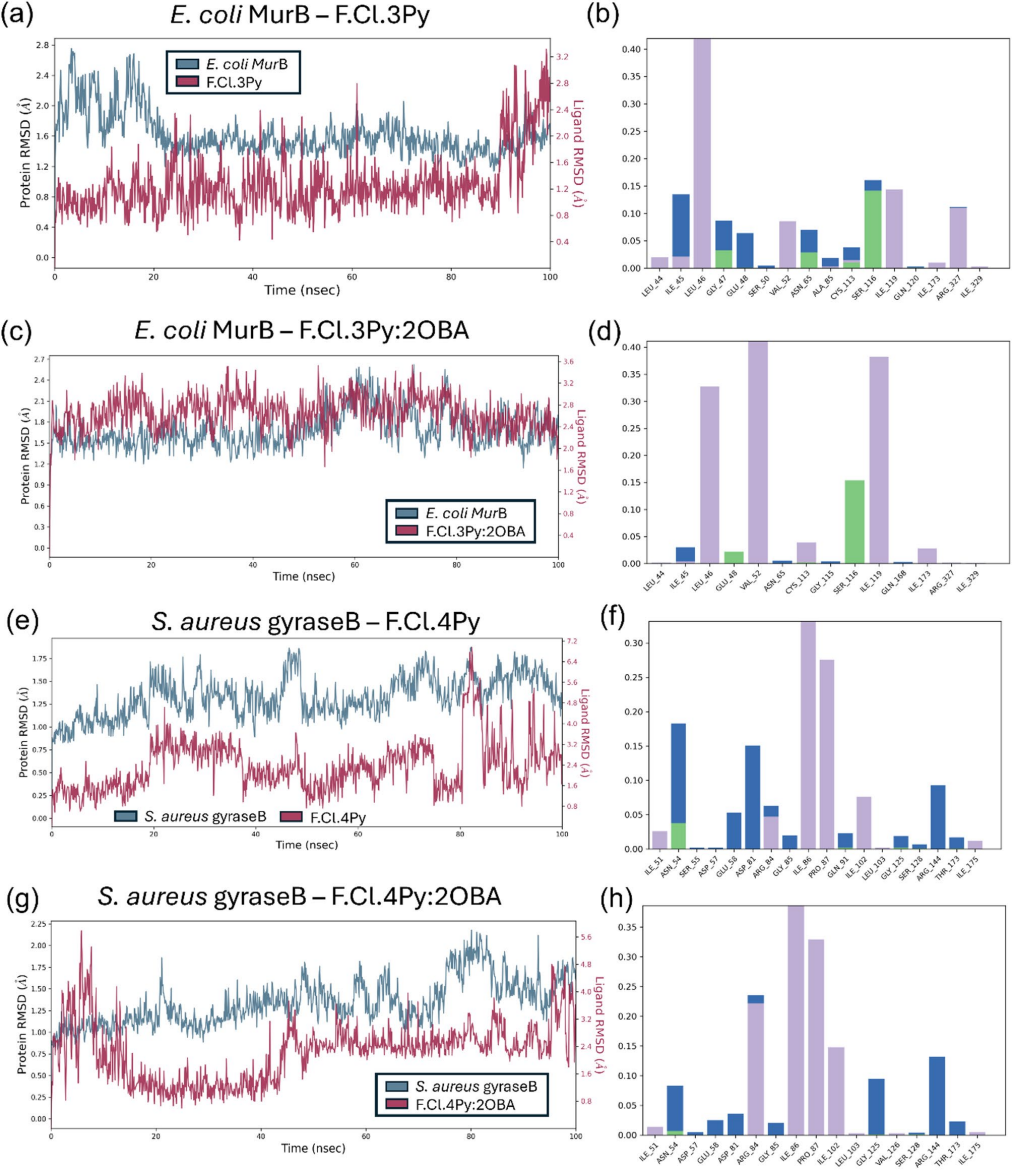
Analysis of RMSD and protein-ligand interactions of 3Py, 4Py and their corresponding HB complexes from MD simulation trajectories. (**a**) RMSD plot and (**b**) molecular interactions of 3Py (denoted as F.Cl.3Py in fig) (**c**) RMSD plot and (**d**) molecular interactions of HB complex of 3Py:2OBA (denoted as F.Cl.3Py:2OBA in fig) within the active site of *E. coli* MurB, (**e**) RMSD plot and (**f**) molecular interactions of 4Py (denoted as F.Cl.4Py in fig) and (**g**) RMSD plot and (**h**) molecular interactions of HB complex of 4Py:2OBA (denoted as F.Cl.4Py:2OBA in fig) within the active site of *S. aureus* GyraseB.

Figure 9 presents the protein-ligand RMSD and the molecular interactions of the Schiff bases with their respective target proteins. For the *E. coli* MurB–3Py complex, the ligand remained highly stable within the catalytic site of MurB (supplementary information, Video S1). Ligand stability is further supported by the protein-ligand RMSD analysis (Fig. 9a). The RMSD of 3Py remains stable, ranging from 0.8 Å to 3.2 Å, with an average RMSD of 1.2 Å during the first 90 ns (Fig. 9a). Minor fluctuations observed between 20 and 25 ns and 40–60 ns are attributed to rotations around the N = CH bond. At approximately 20 ns, a 180º rotation along the bond explains the RMSD variations between 0.8 Å and 2.4 Å. Similarly, between 40 and 60 ns, rotation of the N = CH bond accounts for the observed fluctuations in the ligand RMSD (supplementary information Video S1, Fig. 9a). Beyond 90 ns, a significant fluctuation in RMSD is observed, likely due to a temporary displacement of the ligand from its docked position before returning to its original conformation. Overall, the N = CH bond of the pyridine moiety caused the ligand RMSD fluctuation. The molecular interactions of the ligand throughout the MD simulation are illustrated in Fig. 9b. The ligand primarily engages in hydrophobic interactions, followed by HB interactions and water-bridged H-bonds. Key residues contributing to hydrophobic interactions include Leu46, Val52, Ile119, and Arg327, while Leu44, Ile45, and Ile173 play a minor role in these interactions. H-bonds are formed with Gly47, Asn65, Cys113, and Ser116, whereas water-bridged H-bonds involve Ile45, Gly47, Glu48, Asn65, Ala85, Cys113, and Ser116 (Fig. 9b). With respect to its corresponding HB complex, 3Py and 2OBA remain stable throughout the simulation period (supplementary information Video S2). The protein-ligand RMSD plot obtained for the complex is represented in Fig. 9c. For a simulation of 100 ns, the ligand RMSD is obtained between 2.0 Å and 2.8 Å with intermittent RMSD fluctuations observed at ~ 20, 40, 56 and 70–80 ns (Fig. 9c). The RMSD fluctuation observed within the first 20 ns is due to a slight adjustment of 2OBA in the active site. The minor fluctuation obtained at 40–50 (3.2 Å), 56 ns (1.6 Å–3.2 Å) and 70–80 ns (3.2 Å) are due to the flexibility observed in the alkyl chain of 2OBA (Fig. 9c). This suggests the synergistic action of both the complexes. The molecular interactions for the HB complexes are shown in Fig. 9d. Leu46, Val52, Cys113, Ile119, and Ile173 are involved in hydrophobic interactions while Glu48 and Ser116 are involved in H-bond interactions. Among the two residues, Ser116 is majorly involved in H-bond (Fig. 9d).

In the active site of *S. aureus* GyraseB, 4Py exhibits a ligand RMSD ranging from 0.8 Å to 6.8 Å. Supplementary information Video S3 illustrates the dynamics of 4Py within the active site of GyraseB. For most of the simulation time, the ligand RMSD fluctuates between 0.8 Å and 4.0 Å (Fig. 9e). The ligand remains well-anchored in the active site, with flexibility primarily observed in the N = CH bond and changes in ligand orientation relative to the pyridine moiety. These fluctuations in the orientation of pyridine moiety account for the RMSD increase from 0.8 Å to 1.6 Å to 1.6 Å–3.2 Å at approximately 20 ns (Fig. 9e). A major ligand fluctuation (0.8 Å – 6.4 Å) observed at 80 ns and beyond indicates that the ligand reorients by 90º relative to its initial position. This reorientation is confined to the pyridine moiety. The subsequent decrease in RMSD suggests that the ligand attempts to return to its original binding conformation (supplementary information Video S3). The molecular interactions between the ligand and active site residues post-MD simulation are illustrated in Fig. 9f. The predominant interactions include hydrophobic interactions and H-bonds via water bridges. Ile51, Arg84, Ile86, Pro87, Ile102, and Ile175 contribute to hydrophobic interactions, while Asn54, Glu58, Asp81, Gly85, Gln91, Gly125, Ser128, Arg144, and Thr173 participate in H-bonds via water bridges (Fig. 9f). The interaction of the HB complex of 4Py and 2OBA within the active site of *S. aureus* GyraseB is shown in supplementary information Video S4. Within the active site of *S. aureus* GyraseB, 4Py exhibits stable interaction while 2OBA dissociates from its initial docked position . The ligand RMSD generated for the HB complex ranges from 0.8 Å to 4.8 Å (Fig. 9g). For the initial 10 ns, the ligand RMSD fluctuates between 0.8 Å and 5.6 Å, then from 20 to 40 ns the RMSD remains stable between 0.8 and 1.6 Å. For the simulation time from 45 to 100 ns the RMSD remained quite stable between 2.4 Å and 3.4 Å and in the final 5 ns, the RMSD showed a large fluctuation between 2.4 Å and 4.8 Å (Fig. 9g). The fluctuations observed in this case are due to the dissociation of 2OBA from the docked site and minor adjustments of 4Py within the active site. Therefore, it is observed that the displacement of one ligand from the active site into the solvent during the MD simulation suggests transient interactions, that may affect the antimicrobial activity of the compound compared to its Schiff base, 4Py. This aligns with the experimental analysis that the HB complexes showed lower antimicrobial activity in *S. aureus* compared to 4Py. The molecular interactions of the HB complex are shown in Fig. 9h. Hydrophobic interactions predominate throughout the simulation, followed by H-bonds through water bridges. Ile51, Arg84, Ile86, Pro87, Ile102 contribute towards hydrophobic interactions whereas Asn54, Glu58, Asp81, Gly85, Gly125, Arg144, and Thr173 are involved in H-bonds via water bridges.

In the RMSD plots (Fig. 9), the protein backbone is shown in blue, while the ligand RMSD is represented in maroon. Molecular interactions are colour-coded as follows: H-bonds in green, water bridges in blue, and hydrophobic interactions in purple.

Overall, both the compounds 3Py and 4Py (Schiff bases) exhibited stable interactions within their respective target active sites, with flexibility primarily observed around the N = CH bond. 3Py demonstrated a highly stable interaction within the active site of *E. coli* MurB, maintaining its position throughout the simulation with minimal fluctuations. The observed variations were primarily due to rotational movements around the N = CH bond, which briefly altered the orientation of the pyridine moiety without significantly affecting the overall binding stability. Similarly, 4Py remained well-anchored within the active site of *S. aureus* GyraseB, showing a comparable degree of stability despite some fluctuations. The ligand underwent a 90º orientation of its pyridine moiety at around 80 ns, it returned to its original orientation, suggesting a dynamic but stable interaction. However, compared to the 3Py and 4Py (Schiff bases), their corresponding HB complexes showed lower stability, when 4Py form HB complex with the 2OBA viz., 4Py:2OBA dissociating from the active site. This correlates with lesser the antimicrobial activity obtained for the HB complexes compared to their 3Py and 4Py experimentally.

#### **ADME/T analysis and drug-likeness properties**

Drug-likeness properties i.e., absorption, diffusion, metabolism, excretion and toxicity (ADME/T) and their analysis are crucial parameters for assessing a compound’s potential as a drug candidate. These properties can be evaluated using online tools such as SwissADME^56^. The drug-likeness characteristics of streptomycin, Schiff bases, and 2OBA are illustrated in supplementary information Fig. 9 and summarized in supplementary information Table S3. For a compound to be considered ideal, its physicochemical properties should fall within the pink hexagon in the bioavailability radar image . Streptomycin exhibits favourable drug-like properties for most parameters except molecular weight and polarity (supplementary information Fig. 9a). In contrast, the 3Py and 4Py Schiff bases deviate from the optimal range only in terms of in-saturation (supplementary information Fig. 9b). 2OBA also exhibits a slight deviation from the optimal range for in-saturation (supplementary information Fig. 9c). One of the key criteria for determining oral bioavailability is Lipinski’s Rule of Five (RO5). The 3Py and 4Py and 2OBA passed RO5 without any violations, whereas streptomycin three violations viz., molecular weight, and the number of H-bond donors and acceptors. Additionally, the compounds were evaluated against Ghose, Veber, Egan, and Muegge rules, as summarized in supplementary information Table S3. Overall, 3Py, 4Py, 2OBA, and the standard antibiotic streptomycin exhibit drug-likeness characteristics, indicating their potential as drug candidates.

## Conclusions

The influence of position of pyridyl nitrogen and its HB interactions with *n*OBAs on antibacterial activity has been studied against *S. aureus* and *E. coli* by in vitro and in silico techniques. The ADME/T studies reveal that the 3Py and 4Py have been passed Lipinski’s rule of five without any violations and they have drug-likeness properties. Interestingly, the position of pyridyl nitrogen significantly influenced on antibacterial activity. The pyridyl nitrogen atom at the 4th position (4Py) of the Schiff base showed more inhibition towards *S. aureus*. In contrast to it, the pyridyl nitrogen at the 3rd position (3Py) of the Schiff base showed more inhibition towards *E. coli*. These observations are also supported by molecular docking and simulation analysis as 4Py is studied to be well-anchored within the active site of *S. aureus* GyraseB and 3Py has high and stable interactions within the active site of *E. coli* MurB. Both 3Py and 4Py exhibited higher antimicrobial activity than the *n*OBAs and their HB complexes against *S. aureus* and *E. coli.* From the molecular dynamic simulations, it is revealed that the HB interactions are dissociating in HB complexes at the docking site during which the pyridine moiety fluctuates and is intact with the active site whereas the alkyloxybenzoic acid is moving into the solvent, because of it the complexes have lower antibacterial activity.

The in vitro studies revealed that the HB complexes viz., 3Py:*n*OBA and 4Py:*n*OBA have higher antimicrobial activity than the corresponding *n*OBAs against *S. aureus*, conversely the complexes showed lower activity against *E. coli*. As the alkyl chain length increases, antibacterial activity is found to decrease against *S. aureus* and *E. coli* in the present studies.

The in silico analysis of Schiff bases (3Py and 4Py) and the HB complexes against *E. coli* and *S. aureus* protein targets demonstrates a strong correlation with the experimental results, supporting the potential of these compounds as effective antimicrobial agents. This correlation between computational and experimental results may reinforce the design of novel antimicrobial agents, in future. Moreover, these findings emphasize the critical need to explore novel antimicrobial compounds in the ongoing effort to combat antimicrobial resistance, which remains a significant global health challenge.

## Electronic supplementary material

Below is the link to the electronic supplementary material.

Supplementary Material 1

Supplementary Material 2

Supplementary Material 3

Supplementary Material 4

Supplementary Material 5

## Data Availability

The datasets used and /or analyzed during the current study available from the corresponding author on reasonable request.
