# Supplementary material for: Influence of pyridyl nitrogen’s position and hydrogen bonding interactions on antibacterial activities investigated by in vitro and in silico
Source: Sci Rep. 2025 Jul 30;15:27816. doi: 10.1038/s41598-025-09049-0 (PMC12311143; doi:10.1038/s41598-025-09049-0)
Supplement: Supplementary file 5 — Supplementary Material 5 [file 41598_2025_9049_MOESM5_ESM.docx]

**Influence of Pyridyl Nitrogen’s position and Hydrogen Bonding interactions on Antibacterial activities investigated by *in vitro* and *in silico***

Suma Ramachandra Gopady^1,2^, Thripthi Nagesh Shenoy^3^, Abdul Ajees Abdul Salam^3,*^, Srikrishna Herga Damodar Kedlaya^2^, Shashanka Puranika Kota^2^ Anusha Prabhu^4^, Yegneswaran Prakash Peralam^5,*^, and Srinivasulu Maddasani^1,^*

^1^Department of Chemistry, Manipal Institute of Technology, Manipal Academy of Higher Education, Manipal – 576 104, Karnataka, India

^2^Department of Applied Biosciences, Bhandarkars’ Arts and Science College, Kundapura, Karnataka, India

^3^Manipal Institute of Applied Physics, Manipal Academy of Higher Education, Manipal – 576104, Karnataka, India

^4^Centre for Microfluidics, Biomarkers, Photoceutics and Sensors (µBioPS), Department of Biotechnology, Manipal Institute of Technology, Manipal Academy of Higher Education, Manipal – 576 104, Karnataka, India

^5^Department of Microbiology, Kasturba Medical College, Manipal Academy of Higher Education, Manipal – 576104, Karnataka, India

Email: ^1^s.maddasani@manipal.edu; ^3^abdul.ajees@manipal.edu; ^5^prakash.py@manipal.edu


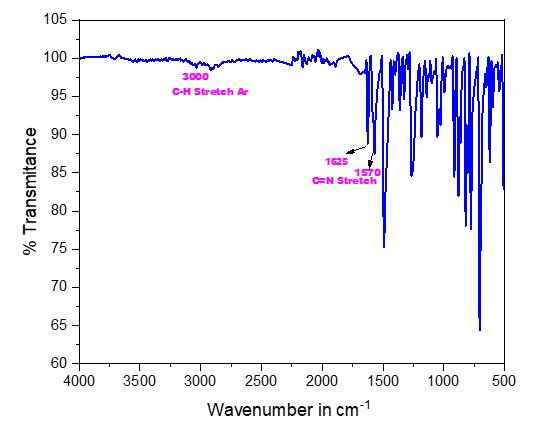


Fig. S1 : FTIR spectrum of 3Py

In the FTIR spectrum of the Schiff base, 3Py, the absorption bands above 3100 cm^-1^ are absent, which confirms the involvement of -NH_2_ group of 3-chloro-4-fluoroaniline in Schiff base formation (Supplementary Fig S1). The absorption band corresponding to C=O stretching of pyridine carboxaldehyde in the range 1715–1695 cm^-1^ is also absent, which indicates the condensation between the aniline and carboxaldehyde. The C–H stretching of aromatic ring and pyridine are noticed at around 3000 cm^-1^. A peak at 1625 cm^- 1^ infers the C=N stretching of Schiff base while the C=N stretching of pyridine moiety is observed by a peak at 1570 cm^-1^.


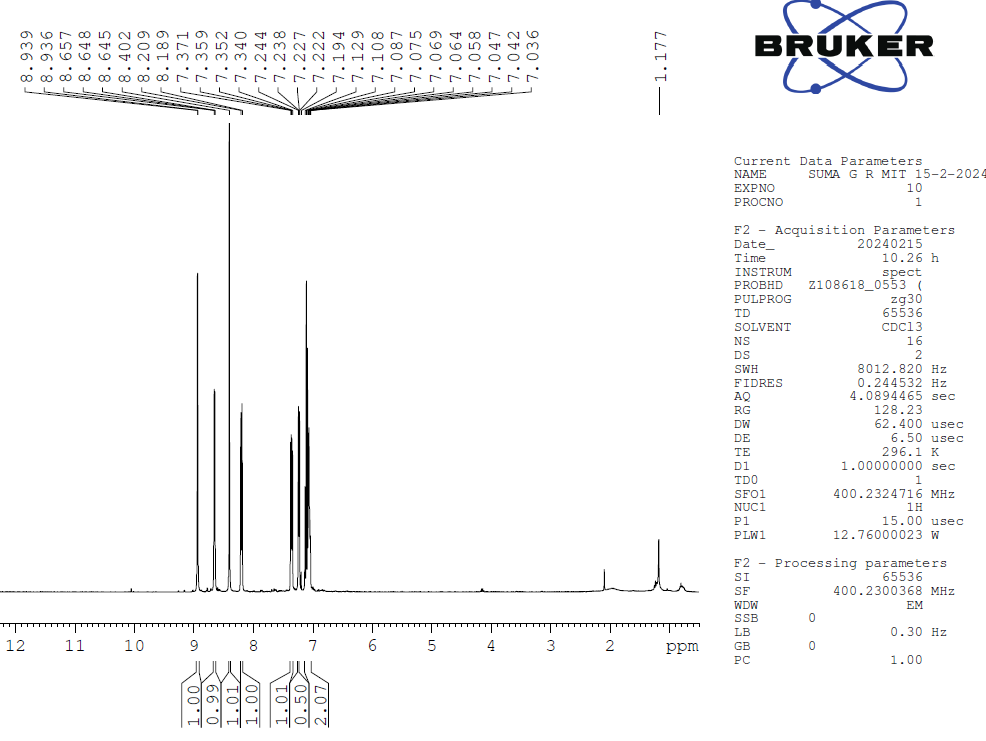


Fig. S2: ^1^H NMR spectrum of 3Py

^1^H NMR (400MHz, CDCl_3_): δ 8.93 (d, *J* = 1.2 Hz, 1H, 1×CH of Pyridine ring), δ 8.65 (t, J_1_= 3.6 Hz, J_2_= 1.2Hz, 1H, 1×CH of pyridine ring), 8.4 (s, 1H, 1×aldimine), δ 8.1 (d, 1H, 1×CH of pyridine ring), 7.35 (q, J =4.8 Hz, 1H×CH of Pyridine ring), 7.24-7.19 (m, 1H, 1×CH of benzene ring), 7.12-7.064 (m, 2H, 2×CH of benzene ring).


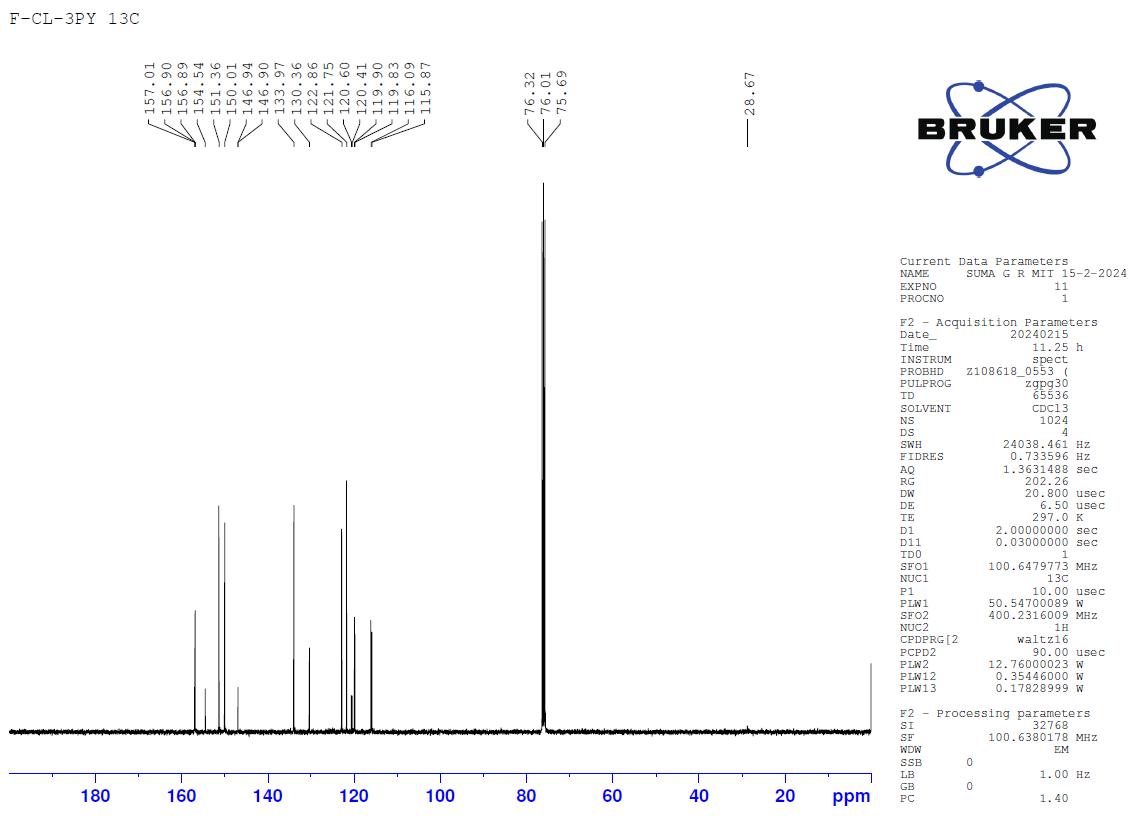


Fig. S3: ^13^C NMR spectrum of 3Py

The observed ^13^C NMR peaks (Supplementary Fig S3) and the values of corresponding chemical shifts for 3Py are: δ 115.98 (1C, s), δ 119.83 (1C, s), δ 120.41(1C, s), 121.75 (1C, s), 122.86 (1C, s), 130.36 (1C, s), 133.97 (1C, s), δ 146.92 (1C, s), 150.01 (1C, s), 151.36 (1C, s), 154.54 (1C, s), 156.93 (1C, s).

**
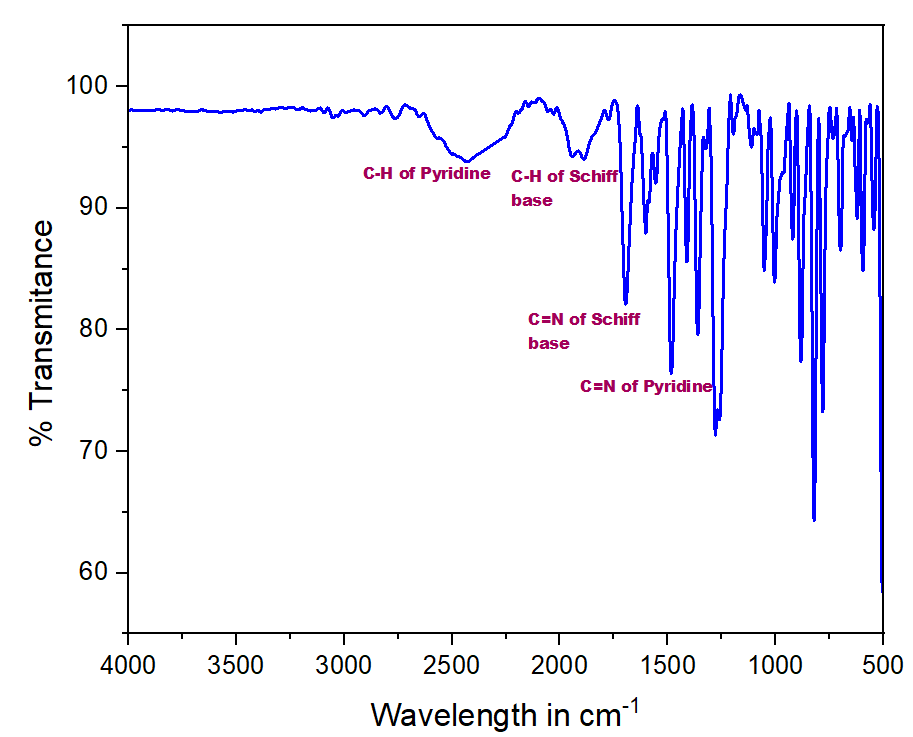
**

Fig.S4 **:** FTIR spectrum of 4Py

The missing of the absorption bands beyond 3100 cm^-1^ in FTIR spectrum (Supplementary Fig S4) recorded for the Schiff base (3) confirms the absence of free -NH_2_ group of 3-chloro-4-methylbenzenamine. The absorption band corresponding to C–O stretching of pyridine carboxaldehyde in the range 1715–1695 cm^-1^ is also absent, which indicates the condensation between the aniline and carboxaldehyde. The C–H stretching of pyridine are noticed at around 2400 cm^-1^. The peak near 1900 infers the C–H stretching of Schiff base. The peak at 1694 cm^-1^ infers the C–N stretching of Schiff base while the C–N stretching of pyridine moiety is indicated by the peak at 1605.70 cm^-1.^


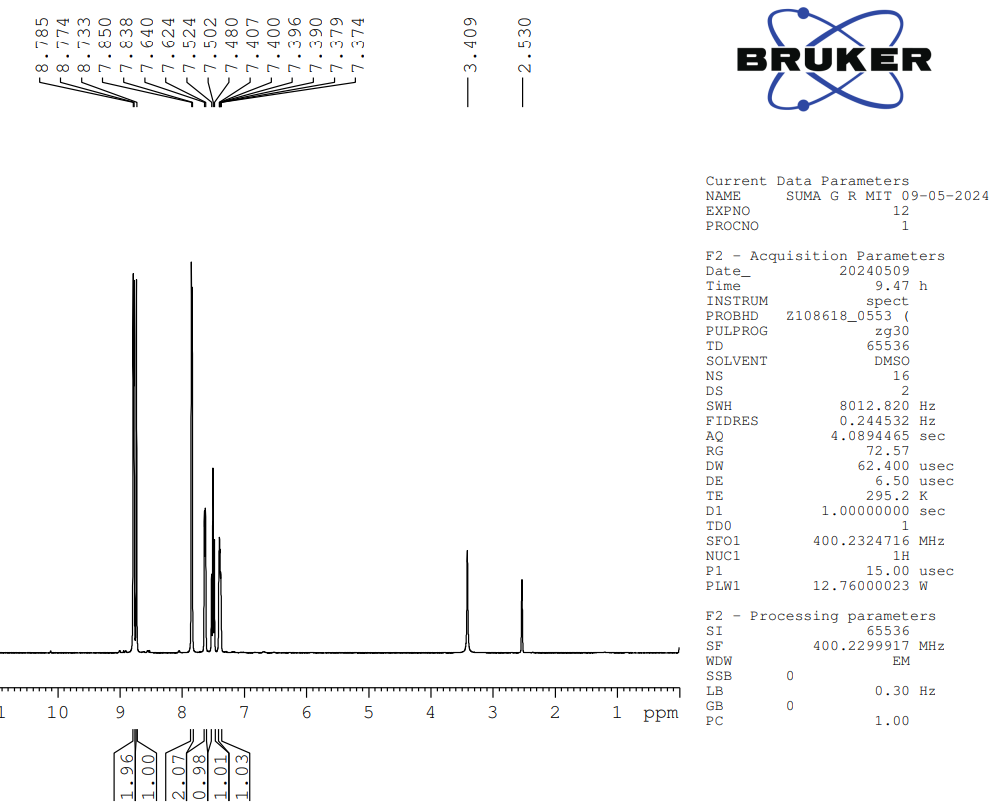


Fig. S5: Proton NMR spectrum of 4Py

^1^H NMR (400MHz, DMSO): δ 8.77 (d, *J* = 3.04 Hz, 2H, 2 × CH of Pyridine ring), 8.73 (s, 1H, 1 × aldimine), δ 7.84 (d, *J*= 4.8 Hz, 2H, 2×CH of pyridine ring), δ 7.63 (d, *J*= 6.4 Hz, 1H, 1 ×CH of benzene ring), 7.502 (t, *J*_1_ =8.8 Hz, *J*_2_ =8.8 Hz, 1H × CH of benzene ring), 7.40-7.37 (m, 1H, 1 × CH of benzene ring).


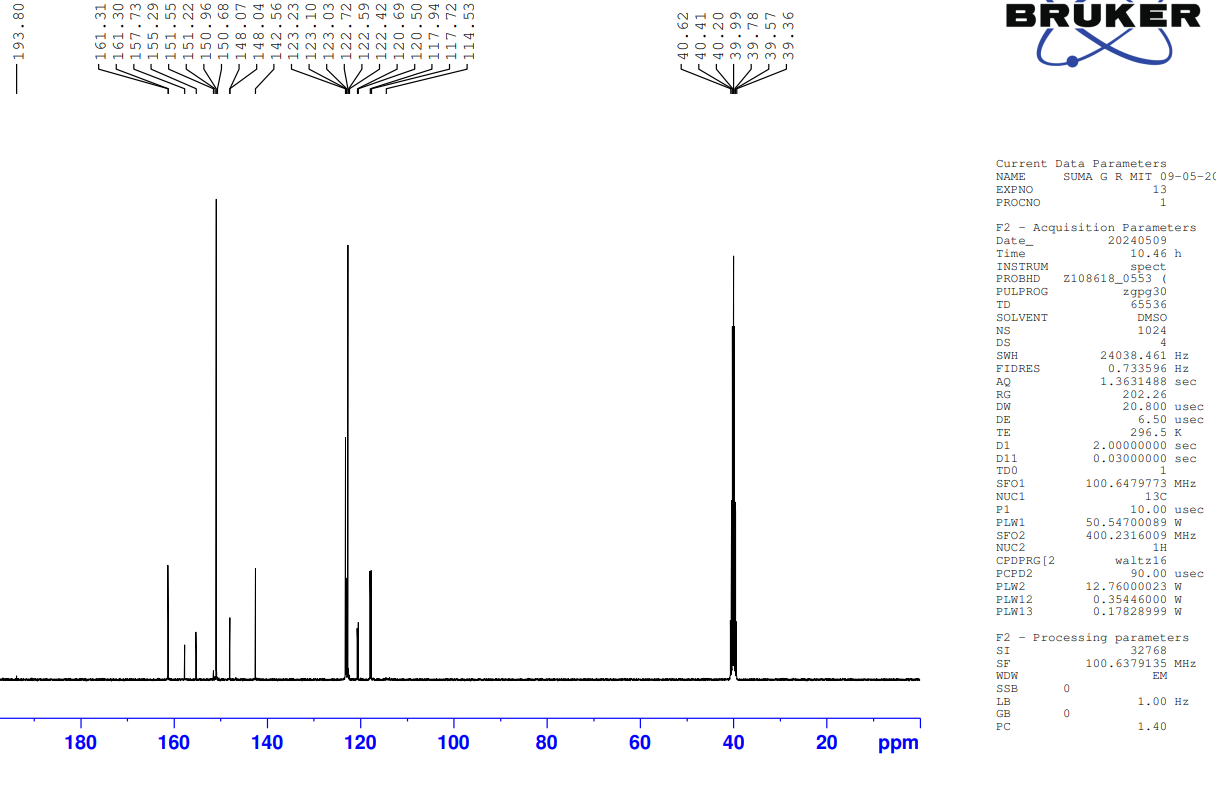


Fig. S6 **:** ^13^C NMR spectrum of 4Py

The observed ^13^C NMR peaks and the values of corresponding chemical shifts for 4Py (Supplementary Fig S6) are: δ 117.83 (1C, s), 120.59(2C, s), 122.73 (1C, s), 123.06 (1C, s), 123.23 (1C, s), 142.05 (1C, s), 148.05 (1C, s), 150.65 (1C, s), 151.38 (1C, s), 155.29 (1C, s), 161.30(1C, s).


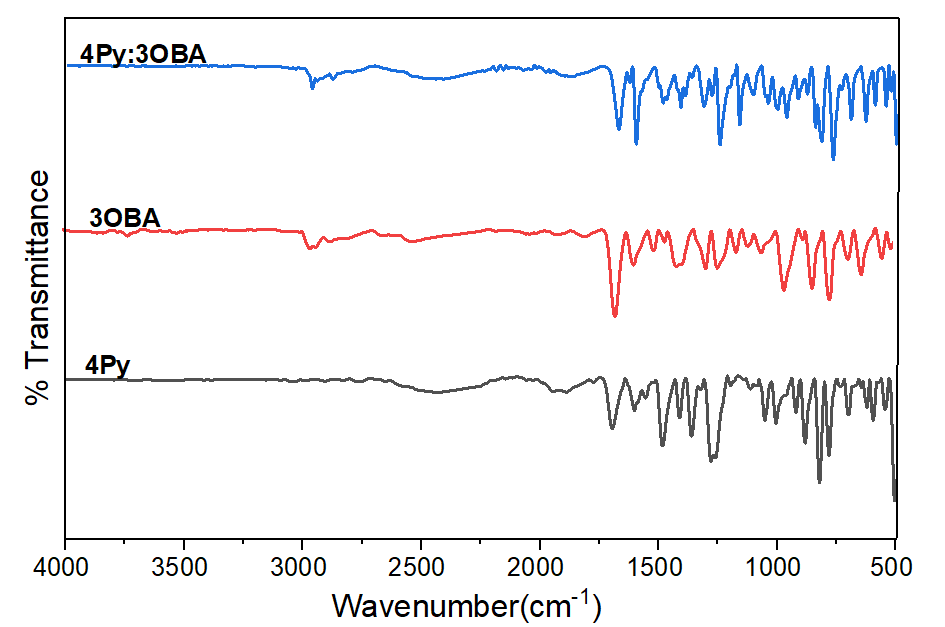


Fig. S7: Comparative FTIR spectra of.4Py, 3OBA and 4Py:3OBA complex


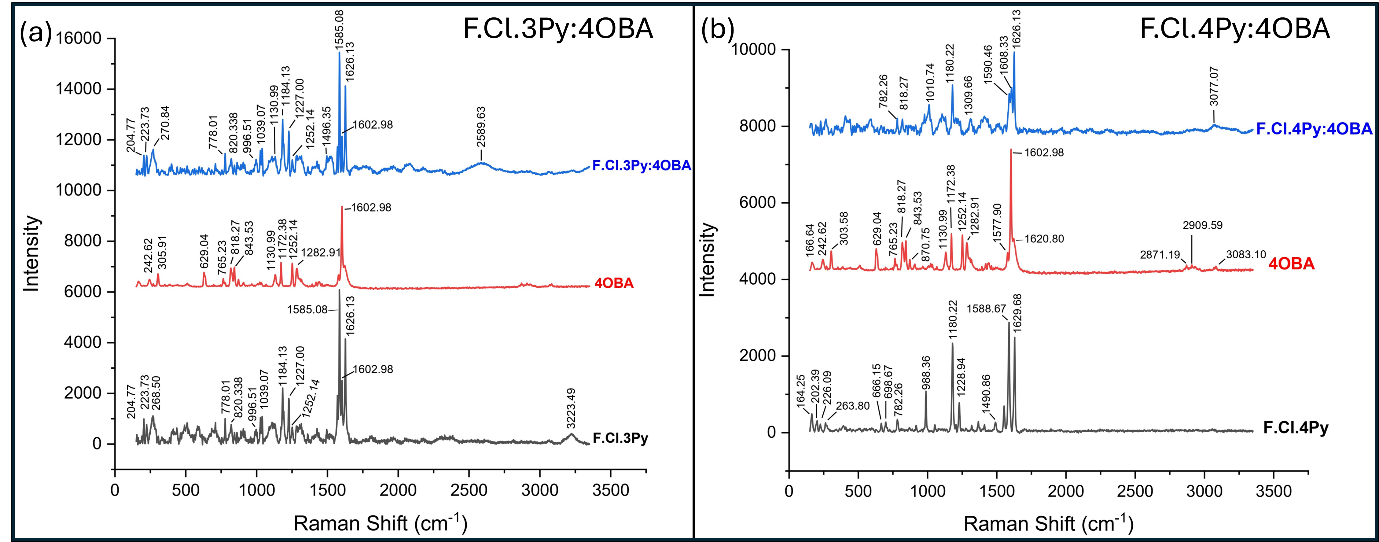


**Fig. S8:** Comparison of Raman spectrum for Schiff bases, *n*-alkyloxybenzoic acid (4OBA), and their respective hydrogen-bonded (HB) complexes for (a) 3Py (shown as F.Cl.3Py), 4OBA and 3Py:4OBA(denoted as F.Cl.3Py:4OBA) and (b).4Py(denoted as F.Cl4Py), 4OBA and 4Py:4OBA(denoted as F.Cl.4Py:4OBA).


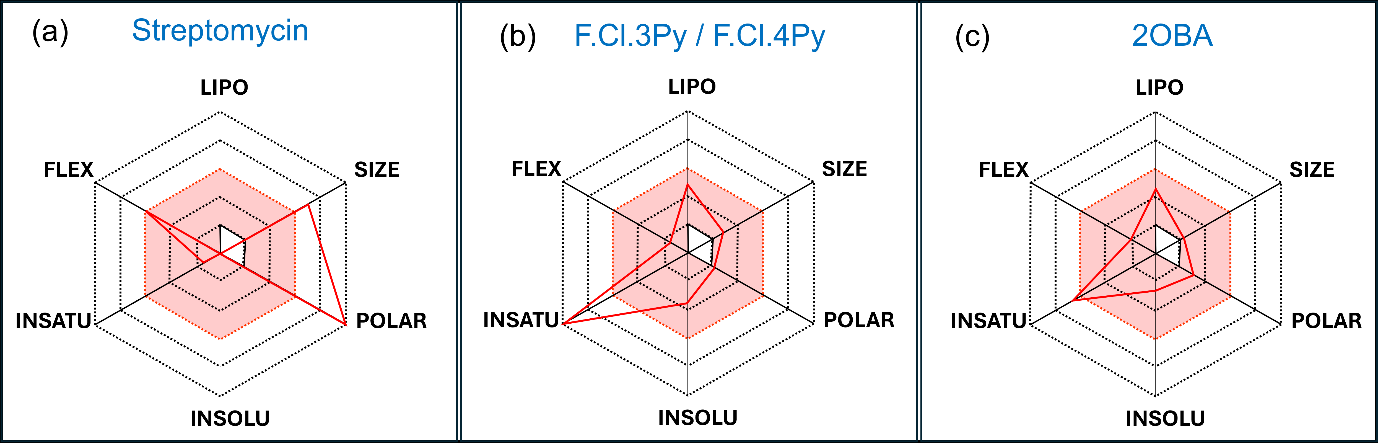


**Fig. S9** Bioavailability radar chart for drug-likeness properties of (a) Standard antibiotic streptomycin, (b) Schiff bases- 3Py/4Py (denoted as F.Cl.3Py / F.Cl.4Py), and (c) 2OBA. The pink region signifies the ideal range for each property. (Lipophilicity: −0.7< XlogP3 < +5.0, size: MW:150 and 500 g/mol, polarity: 20 < TPSA < 130 Å^2^, solubility: log S not greater than 6, saturation: fraction of carbons in the sp^3^ hybridization not less than 0.25, and flexibility: no more than 9 rotatable bonds).

**Table S1:** The frontier molecular orbitals, including the HOMO-LUMO gap, along with other molecular descriptors, determined for the optimized structures of Schiff bases using the def2-SVP basis set. The molecular descriptors were calculated as described previously (Garkusha et al., 2023).

|  | **3Py** | **4Py** |
| --- | --- | --- |
| **Frontier Molecular Orbitals (FMO; eV)** | | |
| E_HOMO_ | -5.893 | -5.866 |
| E_LUMO_ | -3.206 | -3.268 |
| E_LUMO-HOMO_ | 2.687 | 2.598 |
| Ionization Energy (I) = -E_HOMO_ | 5.893 | 5.866 |
| Electron Affinity (A) = -E_LUMO_ | 3.206 | 3.268 |
| **Molecular Chemical Reactivity Descriptors (eV)** | | |
| Global Hardness (η) = (I-A)/2 | 1.3435 | 1.299 |
| Global Softness (δ) = 1/(2η) | 0.3721 | 0.3849 |
| Electronegativity (χ) = (I+A)/2 | 4.5495 | 2.2835 |
| Chemical Potential (µ) = -χ | -4.5495 | -2.2835 |
| Electrophilicity Index (ω) = χ^2^/(2η) | 7.702 | 2.0070 |

**Table S2:** Binding energies (kcal/mol) and molecular interactions of 3Py with *E. coli* MurB and 4Py with *S. aureus* gyraseB and their respective HB complexes with 2OBA. Streptomycin was used as standard antibiotic with both the targets.

| **Target** | **Compound** | **Binding Affinity (kcal/mol)** | **H-bonds** | **Non-bonded Interactions** |
| --- | --- | --- | --- | --- |
| *E. coli* MurB | Streptomycin (Standard) | -4.44 | Glu48, Gly49, Ser50, Pro111, Gln120, Ile173 | Leu44, Ile45, Leu46, Gly47, Asn51, Val52, Gly112, Cys113, Ile119, Gln168, Phe171, Arg327, Ile329 |
|  | 3Py | -7.02 | - | Ile45, Leu46, Gly47, Asn65, Ala85, Gly115, Ile119, Gln120, Ile173 |
|  | 3Py:2OBA | -10.85 | - | - |
| *S. aureus* gyraseB | Streptomycin (Standard) | -6.82 | Asn54, Asp57, Glu58, Asp81, Arg84, Gly85, Gly125, Thr173 | Ala61, Gly83, Ile86, Pro87, Ile102 |
|  | 4Py | -6.20 | - | Glu58, Asp81, Arg84, Gly85, Pro87, Arg144, Thr173 |
|  | 4Py:2OBA | -8.66 | - | - |

**Table S3:** The physicochemical descriptors and Lipinski’s Rule of Five (RO5) used to evaluate the drug-likeness properties of streptomycin, Schiff bases. 3Py and 4Py, and 2OBA.

| **Descriptors** | **Streptomycin**  **(Standard)** | **3Py /**  **4Py** | **2OBA** |
| --- | --- | --- | --- |
| Molecular Weight (MW) | 581.57 | 234.66 | 166.17 |
| TPSA | 336.43 | 25.25 | 46.53 |
| XLogP3 | -7.99 | 2.88 | 2.39 |
| Solubility (Log S) | 2.18 | -3.53 | -2.55 |
| Fraction Csp^3^ | 0.86 | 0 | 0.22 |
| No. of rotatable bonds | 9 | 2 | 3 |
| No. of H-bond acceptors | 15 | 3 | 3 |
| No. of H-bond donors | 12 | 0 | 1 |
| Molar Refractivity | 130.43 | 62.9 | 44.7 |
| Lipinski violations | 3 | 0 | 0 |
| Ghose violations | 4 | 0 | 0 |
| Veber violations | 1 | 0 | 0 |
| Egan violations | 1 | 0 | 0 |
| Muegge violations | 4 | 0 | 1 |

**Legends for video files:**

Video S1: MD simulation of 3Py with *E.coli* MurB protein

Video S2: MD simulation of 3Py:2OBA with *E.coli* MurB protein

Video S3: MD simulation of 4Py with *S. aureus* gyraseB protein

Video S4: MD simulation of 4Py:2OBA with *S. aureus* gyraseB protein

*****
